# Magnesium‐Mediated Electrochemical Synthesis of Ammonia

**DOI:** 10.1002/advs.202504882

**Published:** 2025-05-15

**Authors:** Ishita Goyal, Vamsi V. Gande, Rajan R. Bhawnani, Rebecca Hamlyn, Ahmed A. Farghaly, Meenesh R. Singh

**Affiliations:** ^1^ Department of Chemical Engineering University of Illinois Chicago Chicago Illinois 60607 United States; ^2^ Chemical Sciences Division Lawrence Berkeley National Laboratory Berkeley California 94720 United States; ^3^ Chemical Sciences and Engineering Division Argonne National Laboratory Lemont Illinois 60439 United States; ^4^ Pritzker School of Molecular Engineering The University of Chicago Chicago Illinois 60637 United States; ^5^ Present address: Department of Chemical Engineering Indian Institute of Technology Hyderabad Hyderabad 502285 India

**Keywords:** Calcium‐mediated NH_3_ synthesis, Non‐aqueous electrochemical N_2_ reduction, electrochemical NH_3_ synthesis, Lithium‐mediated NH_3_ synthesis, Magnesium‐mediated NH_3_ synthesis

## Abstract

Metal‐mediated electrochemical synthesis of ammonia (NH_3_) is a promising method to activate N_2_ at room temperature. While a Li‐mediated approach has been optimized to produce NH_3_ at high current density and selectivity, Li's scarcity and its highly negative plating potential limit scalability and energy efficiency. Alternative mediators have been proposed, but only Ca has shown some promise, achieving ≈50% Faradaic efficiency (FE), though requiring voltages beyond −3 V. Here, we report a Mg‐mediated nitrogen reduction reaction (Mg‐NRR), where N_2_ is activated on Mg to form Mg_3_N_2_, followed by protolysis to release NH_3_ and regenerate Mg. A notable NH_3_ FE of 25.28 ± 3.80% is achieved at a current density of −45 mA cm^−2^, corresponding to an NH_3_ partial current density of −11.30 ± 1.77 mA cm^−2^ under 6 bar N_2_. Isotope‐labeled experiments confirm that NH_3_ originates from N_2_, with similar FE (25.15 ± 1.01%). Importantly, NH_3_ production is demonstrated at a total cell potential as low as −3 V. This Li‐free Mg‐NRR system offers key advantages, including lower energy input and use of earth‐abundant materials, making it a scalable route for sustainable NH_3_ synthesis.

## Graphical abstract

This study demonstrates a lithium‐free, magnesium‐mediated electrochemical process for converting nitrogen (N₂) to ammonia (NH₃) under mild conditions. Using abundant materials, the system achieves ≈27% Faradaic efficiency at lower voltages than Li‐ and Ca‐mediated systems. Isotope‐labeling confirms NH₃ production from N₂, showcasing a promising, energy‐efficient route for sustainable ammonia synthesis.

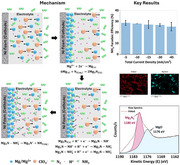

## 1 Introduction

Metal‐mediated ammonia synthesis has garnered significant global attention as the only method capable of activating nitrogen (N₂) at ambient conditions and converting it into ammonia (NH_3_).^[^ ^1^, ^2^, ^3^, ^4^, ^5^, ^6^, ^7^ ^]^ Among the various metal‐mediated approaches, the Lithium (Li)‐mediated nitrogen reduction reaction (Li‐NRR) has been extensively studied and thoroughly explored.^[^ ^8^ ^]^ Remarkable progress has been made, with FEs for NH₃ production reaching nearly 100%, achieved through the use of imide‐based Li salts.^[^ ^9^ ^]^ Additionally, current densities as high as ≈−700 mA cm^−2^ have been reported. Fu et al.^[^ ^10^ ^]^ advanced this method further by developing a continuous flow process, achieving a 61% NH₃ FE via hydrogen oxidation on a Pt‐Au alloy anode.

Despite these advancements, Li‐NRR still suffers from poor energy efficiency, substantially lower than that of the traditional Haber‐Bosch process,^[^ ^3^ ^]^ primarily due to the highly reducing electroplating potential of Li (≈−3.04 V versus SHE).^[^ ^3^ ^]^ Moreover, the long‐term stability of Li‐NRR at higher current densities is compromised due to the electrolyte degradation, which impairs Li recovery, and the high cost of Li salts makes the process economically unfeasible. Selecting a metal with a lower reducing electroplating potential could significantly enhance energy efficiency. These limitations underscore the need to explore alternative metal‐mediated systems that can offer improved stability and cost‐effectiveness.

To explore these alternative mediators, the first step is to understand what makes Li metal effective for ammonia production in a mediated process. The process proceeds with a series of electrochemical and thermochemical steps. The reported mechanism involves Li deposition (electrochemical step) on a cathode followed by N₂ activation on Li to form Li nitride (thermochemical step), followed by its protolysis (thermo/electrochemical step) using a proton (H^+^) donor (e.g., ethanol) to generate NH₃, along with the regeneration of Li sites.^[^ ^5^, ^7^, ^11^, ^12^, ^13^, ^14^, ^15^ ^]^ Based on the unique features of Li including its stable nitride decomposition and optimal solid electrolyte interphase (SEI), which contribute to its exceptional selectivity, several criteria for a successful mediator of NH₃ electrosynthesis have been previously identified,^[^ ^16^, ^17^, ^18^, ^19^, ^20^, ^21^, ^22^, ^23^, ^24^ ^]^ including: (1) spontaneous nitride formation in the presence of N₂ (thermodynamics) and facile N₂ activation (kinetics), (2) stability of surface nitrogen vacancies in the nitride, (3) exergonic binding of N₂ at these surface vacancies, (4) solubility of mediator salts in non‐aqueous electrolytes, and (5) facile diffusion of nitrogen in the bulk nitride.

In our previous work,^[^ ^25^ ^]^ we identified calcium (Ca) and magnesium (Mg) as potential mediators that satisfy these criteria, alongside Li. These metals meet all the aforementioned criteria, with their surface nitride vacancies being more stable than bulk nitride vacancies and exhibiting spontaneous nitride formation at room temperature. Ca (≈−2.87 V versus SHE) has already been investigated in previous work, where it demonstrated favorable and stable performance, particularly using calcium perchlorate tetrahydrate and dimethoxy ethane as the electrolyte. This system yielded 50% FE for NH₃ production. Moreover, Fu et al.'s^[^ ^26^ ^]^ work demonstrated the use of Ca as a mediator for nitrogen reduction in an electrochemical system, achieving a FE of 40 ± 2%. This study verifies Ca's ability to mediate ammonia synthesis in a continuous system, providing a critical step toward utilizing earth‐abundant elements for decentralized ammonia production under ambient conditions. Magnesium is also a promising candidate, as it is more earth‐abundant than lithium and exhibits a lower plating potential than Ca and Li (around −2.37 V versus SHE). These advantages have recently drawn attention to magnesium‐mediated systems to explore their potential for facilitating ammonia synthesis. Preliminary experiments with Mg were also performed in our work previously to show its ability as a successful mediator that achieved a FE of 27%.^[^ ^25^ ^]^ A recent study by Krebz et al.^[^ ^27^ ^]^ also reported NH₃ production in a Mg‐mediated system with an FE of 7% where Mg nitride was synthesized electrochemically and then immersed in an acidic solution to produce ammonia. They utilized a Cu foil electrode and an electrolyte composed of a mixture of Li and Mg salts, running their experiments at 33 °C and 14 bar N_2_ pressure. We, on the other hand, specifically selected ethanol (EtOH) as a weak proton donor, which helps reduce the protonation of Mg, minimizing side reactions and enhancing overall system stability. Our approach involves a more integrated process by using only Mg salts (without Li salts), synthesizing Mg nitride and protonating it to form ammonia within the same cell, while simultaneously regenerating the Mg sites. This simultaneous operation offers potential advantages in simplicity and efficiency for future optimization. The in‐situ Raman characterization presented here offers direct experimental evidence supporting the proposed reaction mechanism. In this work, we systematically investigate magnesium as a mediator for nitrogen activation and ammonia production through control studies, isotope labeling experiments, and variation of current density. Through rigorous experimental studies, we demonstrate that Mg can effectively facilitate nitrogen reduction and serve as a viable catalyst for ammonia synthesis.

## 2 Results and Discussions

**Scheme 1:**
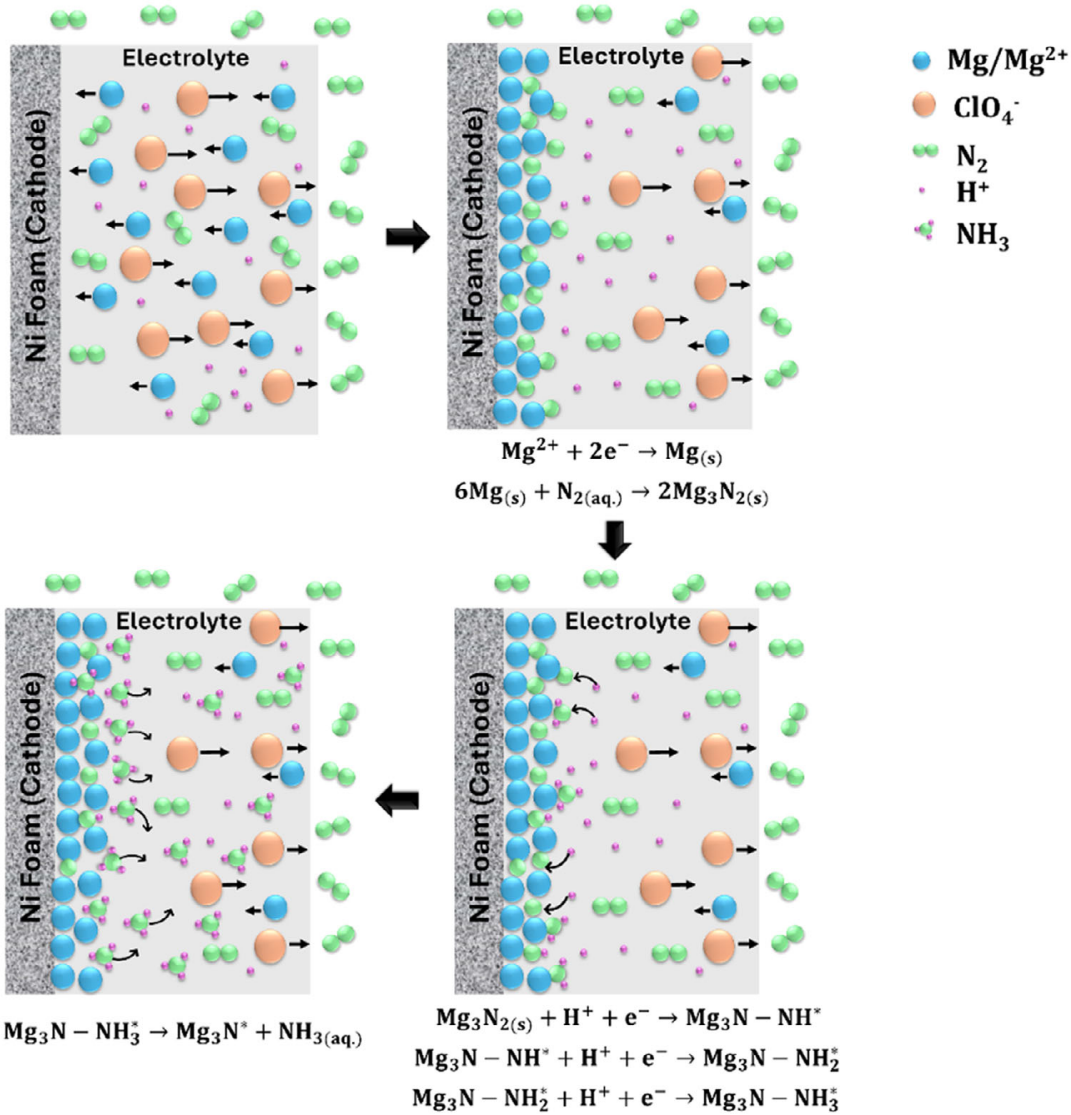
Schematic illustration of Mg‐mediated ammonia synthesis. As shown in the first step, Mg^2^⁺ ions in the electrolyte are reduced at the Ni foam cathode, depositing metallic Mg (Mg⁰). The deposited Mg⁰ then reacts with nitrogen gas (N₂), forming Mg₃N₂. The nature of the solid electrolyte interphase (SEI) plays a crucial role in nitrogen transport and reaction efficiency. If the SEI remains sufficiently porous, it facilitates continuous nitrogen diffusion to the Mg⁰ layer. Subsequently, Mg₃N₂ undergoes protonation through a stepwise mechanism, ultimately yielding ammonia.

With its lower plating potential (−2.37 V versus SHE), Mg has the potential to be a more energy‐efficient alternative. Based on the criteria for successful mediators, Mg‐mediated ammonia synthesis is hypothesized to occur in a manner similar to Li‐ and Ca‐mediated processes, i.e., based on the reaction **Scheme** **1**. As illustrated in the **Scheme**  1, upon the application of a negative current, Mg^2^⁺ ions begin to deposit on the cathode as metallic Mg (Mg⁰). This Mg⁰ subsequently reacts with the available nitrogen, forming Mg nitride (Mg₃N₂) on the cathode surface. The SEI formed during this process plays a crucial role in determining the functionality of the cell. If the SEI is sufficiently porous, it facilitates the transport of nitrogen to react with the Mg⁰ layer. The formed Mg nitride undergoes protonation, releasing ammonia. This process creates nitrogen vacancies within the SEI, allowing another N₂ molecule to react with Mg⁰, thereby sustaining the reaction cycle.

**Figure 1:**
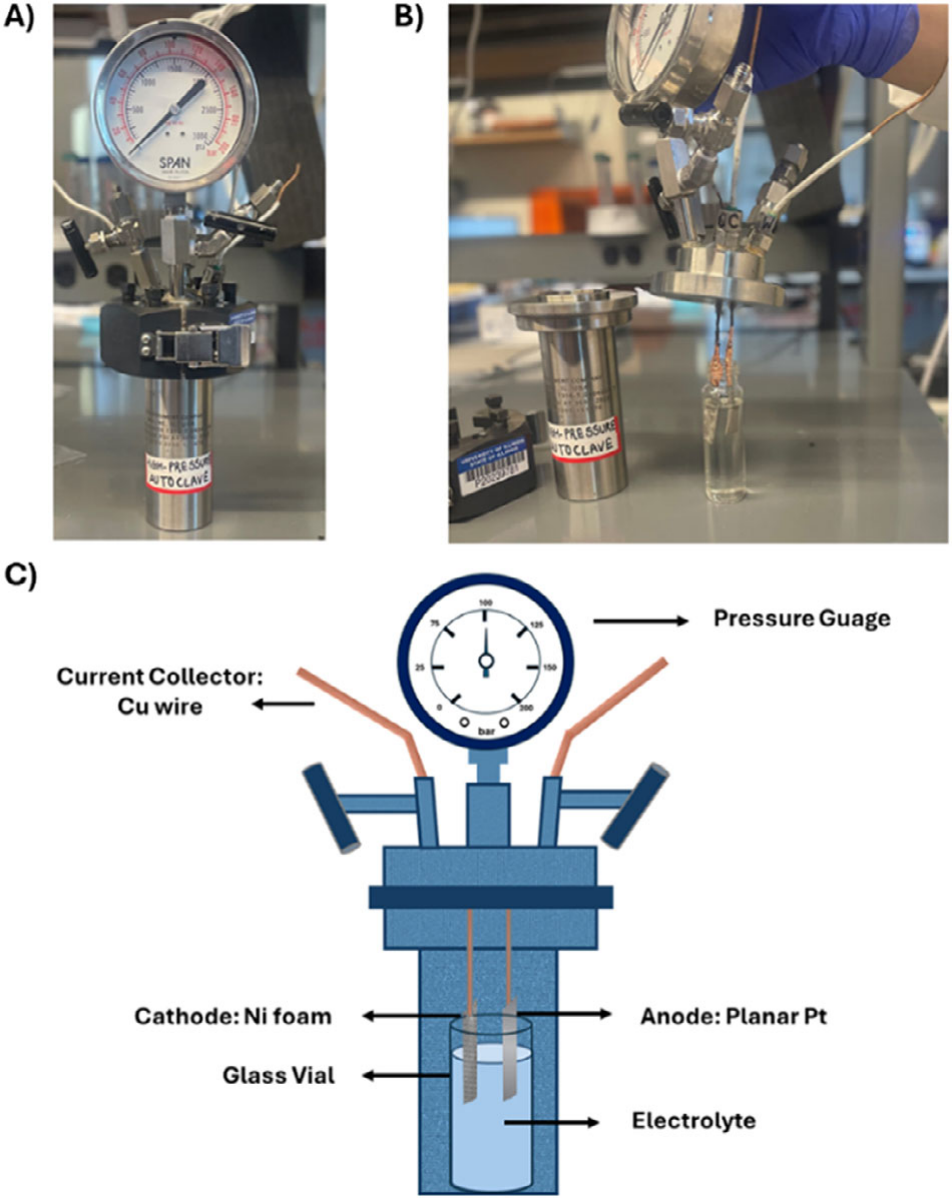
A) Customized autoclave reactor for electrochemical experiments. B) Assembly of electrolyte and 2 electrodes of the electrochemical cell in high pressure autoclave reactor. C) Schematic and configuration of the batch autoclave for electrochemical Mg‐mediated ammonia synthesis.

In our previous investigation on Ca‐ and Li‐mediated ammonia synthesis,^[^ ^3^, ^25^ ^]^ we have identified an optimal concentration of the H^+^ donor and N_2_ pressure crucial for achieving a balance between Li_3_N protolysis and Li protolysis steps. Similarly, for Mg‐mediated NH_3_ synthesis, we chose to operate the reactor at a N_2_ pressure of 6 bars. Ethanol (EtOH, 0.065 M) was designated as the proton (H^+^) donor. To accommodate the high pressures required for the process, we implemented a modified autoclave setup. A schematic and a a visual representation of the experimental setup are shown in **Figure** **1**. Within our experimental framework, Ni foam was employed as the cathode, complemented by Pt gauze serving as the anode in a membrane‐less configuration. Maintaining a stirring rate of ≈700 rpm ensured effective mixing of the electrolyte throughout the reaction. However, we encountered challenges with the solubility of Mg salts in various solvents, which were tested for Li‐NRR and Ca‐NRR, which proved to be quite limited and difficult to establish.

In metal nitride‐mediated ammonia synthesis, the SEI plays a significant role in determining system performance. The selection of salt, solvent, and alcohol profoundly influences the composition and stability of the SEI, thereby affecting the FE of ammonia production. In Li‐mediated systems, salts such as LiBF_4_,^[^ ^9^ ^]^ LiClO_4_, and imide‐based salts have been effectively utilized, underscoring the critical impact of salt selection on SEI characteristics and its subsequent effect on system stability and ammonia selectivity. The choice of Mg salt was influenced by three key factors: solubility, conductivity, and SEI stability. Mg perchlorate was selected due to its high solubility in organic solvents like propylene carbonate and dimethyl formamide (DMF), as well as its availability in anhydrous form. Its intermediate‐sized perchlorate anion provided a balance between ionic conductivity and SEI stability, addressing the trade‐off between these two properties. This combination ensured effective electrochemical performance with minimal iR losses and sufficient SEI stability for ammonia synthesis. The propylene carbonate system exhibited higher viscosity, leading to increased resistance and, consequently, reduced efficiency. A brief experimental protocol for the propylene carbonate system is provided in the SI. The propylene carbonate system was tested at current densities (switching current density approach was employed) of −2 mA cm^−2^ and −30 mA cm^−2^, but NMR analysis of the post‐electrolysis samples showed no ammonia formation. Additionally, the voltage requirements for this system were extremely high, making it unsustainable for further investigation. The voltage and current data are included in **Figure** S1 (Supporting Information).

**Figure 2:**
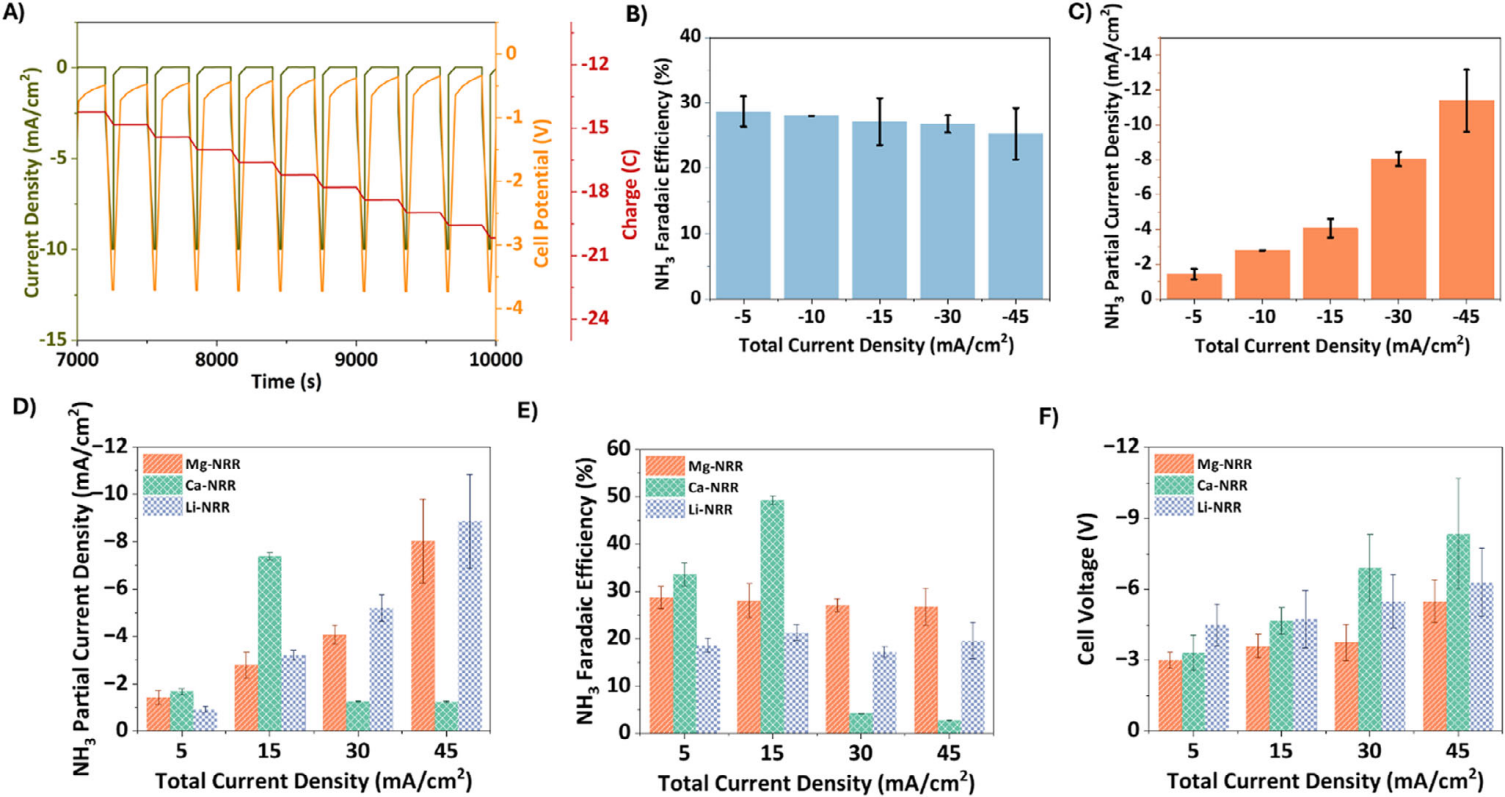
A) Pulsed Chronopotentiometry analysis of Mg‐mediated ammonia synthesis at a current density of −10 mA cm^−2^ (working phase – 1 min) and 0 mA cm^−2^ (resting phase – 4 mins) and working potential range of 3.75–4 V. B) NH_3_ Faradaic efficiencies at different applied current densities. C) NH_3_ partial current densities at different applied total current densities. (Operating conditions: Electrolyte – 0.5 M Mg(ClO_4_)_2_ + 0.065 M EtOH in DMF; 6 bar N_2_ pressure, Ni foam cathode and Pt anode). D) Comparison of NH_3_ partial current density for Li mediated nitrogen reduction reaction (Li‐NRR), Ca‐mediated nitrogen reduction reaction (Ca‐NRR), Mg‐mediated nitrogen reduction reaction (Mg‐NRR).^[^ ^3^, ^29^ ^]^ E) Comparison of NH_3_ Faradaic efficiency for Li‐NRR, Mg‐NRR, and Ca‐NRR.^[^ ^3^, ^29^ ^]^ F) Comparison of cell voltage for Li‐NRR, Mg‐NRR, and Ca‐NRR systems.^[^ ^3^, ^29^ ^]^

Since the propylene carbonate system failed to produce ammonia, the DMF system was further investigated using an electrolyte comprising 0.5 M Mg perchlorate in DMF with 0.065 M EtOH. To stabilize the SEI, we implemented the current switching strategy in this system, similar to the propylene carbonate system following Andersen et al.’s recommendation,^[^ ^28^ ^]^ with 1 min of working time and 1 min of resting time (depending on the applied current density). The duration of the resting time varied and was adjusted as needed to maintain system stability, resulting in variable total runtimes for each current density. The resting current density was maintained at 0 mA cm^−2^ (i.e., at open circuit voltage), while different working current densities were applied. Representative pulsed chronopotentiometry for one applied current density of −10 mA cm^−2^ is shown in **Figure** **2**A (full pulsed chronopotentiometry is shown in **Figure** S2, Supporting Information). The switching time is crucial for stabilizing cell voltage by limiting the growth of the SEI and preserving its stability. During the working time, when a negative current density is applied, Mg deposition primarily occurs. The rate‐limiting steps during this phase include Mg nitridation, Mg_3_N_2_ protolysis, and Mg protolysis.^[^ ^3^ ^]^ Conversely, during the resting time, when the current density is zero, Mg deposition ceases, and thermochemical nitridation and protolysis steps become dominant.^[^ ^5^, ^7^, ^11^, ^13^, ^14^, ^15^ ^]^ The extent of Mg nitridation is contingent upon the N_2_ pressure, while Mg_3_N_2_ protolysis and Mg protolysis are influenced by the concentration of the H^+^ donor (EtOH). In our prior work on Li‐mediated ammonia synthesis,^[^ ^3^ ^]^ we observed that there are two distinct regimes: a H^+^‐limited regime and an N_2_ mass transfer‐limited regime. When operating at lower N_2_ pressures, N_2_ availability becomes relatively low, whereas protons are abundant in the solution. According to the proposed mechanism, under these conditions, N_2_ mass transfer controls the rate of ammonia production. Conversely, at higher pressures, where N_2_ is abundant for nitridation but the same amount of H^+^ donor is used, protonation becomes the limiting factor for ammonia production. The reaction appears to proceed through two interconnected cycles: the nitridation loop and the protonation loop, as shown in **Figure** S3 (Supporting Information). Optimizing the frequency of these loops is critical for achieving the highest ammonia selectivity.

We propose that the formation of the passivating SEI requires time to develop, approximately one hour under the present conditions, and selectively permits the passage of Mg^+2^, N_2_, H_2_, NH_3_, and H^+^.^[^ ^3^ ^]^ In metal‐mediated systems, the oxidation of DMF at the anode likely leads to the formation of a complex SEI composed of various organic and inorganic decomposition products. This SEI layer is critical for protecting the electrode surface and ensuring long‐term system performance by being selectively permeable to Mg ions while minimizing further reduction reactions. The formation of the SEI may involve mechanisms such as oxidation reactions of organic solvents, similar to those observed in related systems like tetrahydrofuran (THF),^[^ ^11^ ^]^ which undergo analogous degradation processes. SEI can influence the transport of both Mg^2^⁺ and N₂. The SEI can act as a diffusion barrier for Mg^2^⁺ ion transport, depending on its composition and thickness, thereby influencing magnesium deposition and overall reaction kinetics. Likewise, a dense or highly resistive SEI could impede N₂ diffusion to reactive sites, potentially influencing nitride formation. Switching times also impact mass transport, as it has been experimentally observed that during resting time, the open circuit potential should be maintained close to ≈−0.1 V to −0.5 V, depending on the current density. This condition is crucial for maintaining a stable and porous SEI, which facilitates the efficient mass transport of N₂ to the fresh Mg layer, enabling its nitridation. Without a stable and permeable SEI, nitrogen would be unable to penetrate and react with the deposited Mg, compromising the overall reaction process. Deviations from ≈−0.1 V to −0.5 V favor H_2_ formation through Mg protolysis. Thus, optimizing the resting time is crucial. Stirring speed also affects Mg_3_N_2_ and Mg protolysis and may vary based on the size and type of stirrer employed.^[^ ^3^ ^]^ If the open circuit potential deviates significantly, adjustments can be made to restore it as briefly described in SI. Lowering the resting time, working time, and stirring speed can bring the potential back to −0.1 to −0.5 V if it drops below this value. Conversely, increasing the resting time, reducing the working time, and enhancing the stirring speed can restore the potential to −0.1 to −0.5 V if it rises above this threshold. Based on our observations, we found that a working time of 1 minute and a resting time of 1 to 6 minutes, depending on the total current density, are optimal for stabilizing the SEI.

When the current density increases, the NH_3_ current densities rise while the NH_3_ FEs remain constant at an average value of ≈27% as shown in **Figure** 2B. The loss in FE could be attributed to excess Mg deposition, electrolyte breakdown, and corrosion. **Figure** 2B,C (**Figure** S4, Supporting Information) demonstrates that the production rates of NH_3_ and H_2_ increase proportionally with rising current density. This suggests that the ratio of Mg protolysis to Mg_3_N_2_ protolysis rates is independent of potential, or perhaps both protolysis steps are barrierless at high potentials. Therefore, the potential‐independent behavior could be due to a fixed fractional coverage of Mg_3_N_2_ on the cathode since protolysis is a thermochemical step. The coverage of Mg_3_N_2_ is balanced by nitridation and protolysis reactions, which are directly related to N_2_ pressure and H^+^ donor, respectively. Moreover, varying Mg deposition rates at higher applied potentials could also influence this behavior. Increased overpotentials may alter Mg electrodeposition morphology, which could in turn affect nitridation kinetics by modifying the accessibility of active sites for N_2_ activation. This phenomenon is closely tied to the stability and properties of the solid‐electrolyte interphase. If Mg deposition occurs rapidly over freshly plated Mg without sufficient time for nitrogen incorporation, the inner Mg layers may become effectively shielded from nitridation. This highlights the importance of optimizing SEI characteristics (e.g., ionic conductivity, N_2_ permeability) to ensure both efficient Mg deposition and sustained nitrogen access to the active metal surface.

**Figure** 2D–F presents a comparison of FEs, ammonia current density, and total cell voltage for Li‐NRR, Ca‐NRR, and Mg‐NRR. The data for Li and Ca have been taken from our previous work for this comparison.^[^ ^3^, ^25^ ^]^ For Mg and Li, we observed that an increase in total current density correlates with a proportional increase in ammonia production rate, while the FE for ammonia remains constant. This indicates that the NH_3_ FE is independent of the applied current densities and the total cell potential for the configuration used. This also suggests that the same ammonia FE can be maintained even at higher current densities. Ca‐NRR also shows significant potential with good ammonia selectivity, although further work is needed to enhance both the ammonia current density and FE. Mg‐NRR, while promising, still requires in‐depth research to achieve comparable FEs and current densities. We hypothesize that the higher potential observed for Ca in electrochemical systems could be due to differences in solvent conductivity, with DMF^[^ ^30^ ^]^ exhibiting the highest conductivity, followed by THF^[^ ^31^ ^]^ and DME.^[^ ^32^ ^]^ Additionally, for Li, the higher salt concentration might contribute to the observed trends. Furthermore, DME and DMF demonstrated excellent electrochemical and thermal stability under operating conditions relevant to ammonia synthesis, exhibiting negligible degradation over extended electrolysis durations. In contrast, THF underwent significant decomposition, as evidenced by substantial post‐electrolysis changes in the electrolyte, including increased viscosity, darkening, and the formation of polymeric or tar‐like residues. These observations raise concerns regarding solvent recyclability and economic viability for scale‐up. The stability of DME and DMF, indicated by the absence of discoloration or viscosity change, underscores their suitability as robust solvent systems for sustained electrochemical nitrogen reduction. Salt concentration plays a crucial role in electrochemical performance, as higher concentrations improve ion mobility, reduce solution resistance, and minimize ohmic losses. However, excessively high concentrations can increase solution viscosity, leading to higher resistance and degraded performance. For instance, in our previous work on the LiNRR^[^ ^3^ ^]^ system, using LIBF_4_ at concentrations of 1 M to 3 M significantly improved performance, however, a 4 M concentration caused performance degradation due to increased viscosity. Thus, identifying the optimal salt concentration is therefore critical for achieving the best performance while managing costs, as higher salt concentrations can raise system expenses. In our earlier study on Ca‐mediated NRR,^[^ ^29^ ^]^ the application of 1 M Ca(ClO₄)₂ solution resulted in lower Faradaic efficiency (FE), the 0.5 M concentration was ultimately chosen as optimal due to its balance of performance and practicality. For Li, a 2 M concentration of Li salt proved to be optimal, leading to a slightly lower applied potential compared to Ca systems. In the case of Mg, a 1 M concentration of Mg perchlorate was initially employed in the previous study.^[^ ^25^ ^]^ Due to the crystallization observed in the solution after 24 h, a lower concentration was tested, and a concentration of 0.5 M was found to be optimal. This adjustment prevented salt crystallization and ensured the solution remained well‐mixed even 24 h after the operation was completed. Comparable performance was achieved using 0.5 M Mg perchlorate, which also demonstrated the lowest applied potential while maintaining satisfactory faradaic efficiencies (FEs). Among the three cations (Ca, Mg, and Li), Mg seems to be the most promising mediator for ammonia synthesis. With a 0.5 M concentration of Mg perchlorate, the cell exhibits lower applied potential while maintaining high FEs. Mg shows significant potential for optimization due to its favorable characteristics, such as a lower energy input compared to Li and the ability to achieve relatively high FE under certain conditions. These traits suggest that, with further investigation and refinement of the reaction system, Mg could become a promising candidate for efficient and sustainable ammonia synthesis. Furthermore, we observed that the working potential for Mg‐NRR is relatively lower compared to Li‐NRR and Ca‐NRR, which supports the concept of a reduced plating potential for Mg. Notably, with Mg‐NRR, we successfully synthesized ammonia at a working electrode potential as low as 3 V.

Cyclic voltammetry (CV) was conducted in a similar reaction set up to investigate the electrochemical behavior of Mg in a 0.5 M Mg(ClO_4_)₂ electrolyte dissolved in DMF with 0.065 M EtOH as a H^+^ donor. The CV profile, recorded between −4 V and 4 V versus Ag/AgCl at a scan rate of 10 mV s^−1^, exhibits distinct cathodic and anodic features corresponding to Mg deposition and stripping processes, respectively, as shown in **Figure** S5 (Supporting Information). On the cathodic sweep, a significant current onset at ≈−2.45 V versus Ag/AgCl indicates the reduction of Mg^2^⁺ to metallic Mg on the working electrode. During the reverse anodic sweep, an oxidation peak is observed ≈1.45 V versus Ag/AgCl, signifying the stripping of deposited Mg^[^ ^33^ ^]^ back into the electrolyte as Mg^2^⁺.

To identify that the NH_3_ is generated from N_2_ and not due to contaminants, the open circuit control experiments were conducted where no potential was applied, and the solutions were stirred for 2 h with 6 bars of N_2_ pressure. ^1^H NMR spectra of both pre‐electrolyte and post‐electrolyte samples showed no ammonia, as shown in **Figure** S6 (Supporting Information). Another control experiment was performed using argon (Ar) gas to pressurize the reactor instead of N_2_​. This was done to verify that ammonia is from N_2_ but not from solvent decomposition. The reactor was pressurized to 6 bar with Ar, and the reaction was carried out at −15 mA cm^−2^ for 4 h. Post‐electrolyte analysis using ^1^H NMR showed no ammonia peaks, as shown in **Figure** S6 (Supporting Information). An electrolyte sample was prepared and left open in the fume hood to ensure that ammonia was not originating from atmospheric contaminants but was instead being electrochemically synthesized. After 2 h, the sample was analyzed by ^1^H NMR, and it showed no ammonia peaks, as depicted in **Figure** S6B (Supporting Information).

To evaluate the stability of the system in terms of operating potential and overall cell performance, long‐term experiments were conducted over a total duration of 94 h, including 47 h of cumulative working time (1 min of working time followed by 1 min of resting time was used). The system operated under a cyclic chronopotentiometric mode, alternating between a working current density of −5 mA cm^−2^ for 1 min and a resting current density of 0 mA cm^−2^ for 1 min. The cell exhibited smooth operation with stable potential values throughout, demonstrating the robustness and stability of the setup, as shown in **Figure** S7A (Supporting Information). However, this experiment was not designed to evaluate the stability of ammonia synthesis due to the membrane‐less configuration of the cell. In such a setup, synthesized ammonia may be oxidized during extended periods of operation.^[^ ^5^, ^34^ ^]^ The NMR quantification revealed the FE to be ≈16%, which was half of what we observed during the 1‐h working time. This suggests that ammonia undergoes oxidation in the membrane‐less setup.

**Figure 3:**
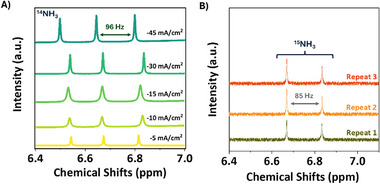
A) ¹H‐NMR spectra of post‐reaction electrolyte solutions collected at varying current densities. The current densities applied are labeled directly within the figure, and the spectra correspond to the ammonia detected in the electrolyte after the electrochemical reaction. B) ¹H‐NMR spectra of Isotope‐labeled post‐reaction electrolytes using ^15^N_2_ performed at −45mA cm^−2^ of current density.

To accurately quantify ammonia and avoid false positives, quantitative analysis of ammonia was conducted using ^1^H NMR spectroscopy, following a previously published protocol that ensures the measured NH_3_​ arises from electrochemical N_2_​ reduction and not from air or other potential contamination sources.^[^ ^1^ ^]^ The triplet peak associated with ammonia containing the most abundant ^14^N_2_ isotope (spin 1 nucleus) appears at 6.83 ppm with a coupling constant of 96 Hz, as shown in **Figure** **3**A. The resulting spectra for the post‐electrolyte samples at different current densities are depicted in **Figure** 3A. Isotope labeling experiments were also performed to ensure that NH_3_ is produced by the electrochemical reduction of N_2_​ and not from contaminants. They were conducted at a current density of −45 mA cm^−2^; this condition resulted in the highest ammonia current density. The isotope‐labeled samples were quantified using ¹H NMR, and the spectra are shown in **Figure** 3B. These experiments yielded a FE of 25.15 ± 1.01%, with an ammonia current of 11.4 mA cm^−2^ at −45 mA cm^−2^. The NMR spectrum of ammonia generated from ¹⁵N₂ displays a characteristic doublet at 6.67 ppm, with a coupling constant of 85 Hz. UV‐vis spectrometry was also used for quantification through the indophenol method. Both UV‐vis and NMR were benchmarked and produced consistent results. The method of additions employed to accurately determine the ammonia concentration is detailed in the Supporting Information.

**Figure 4:**
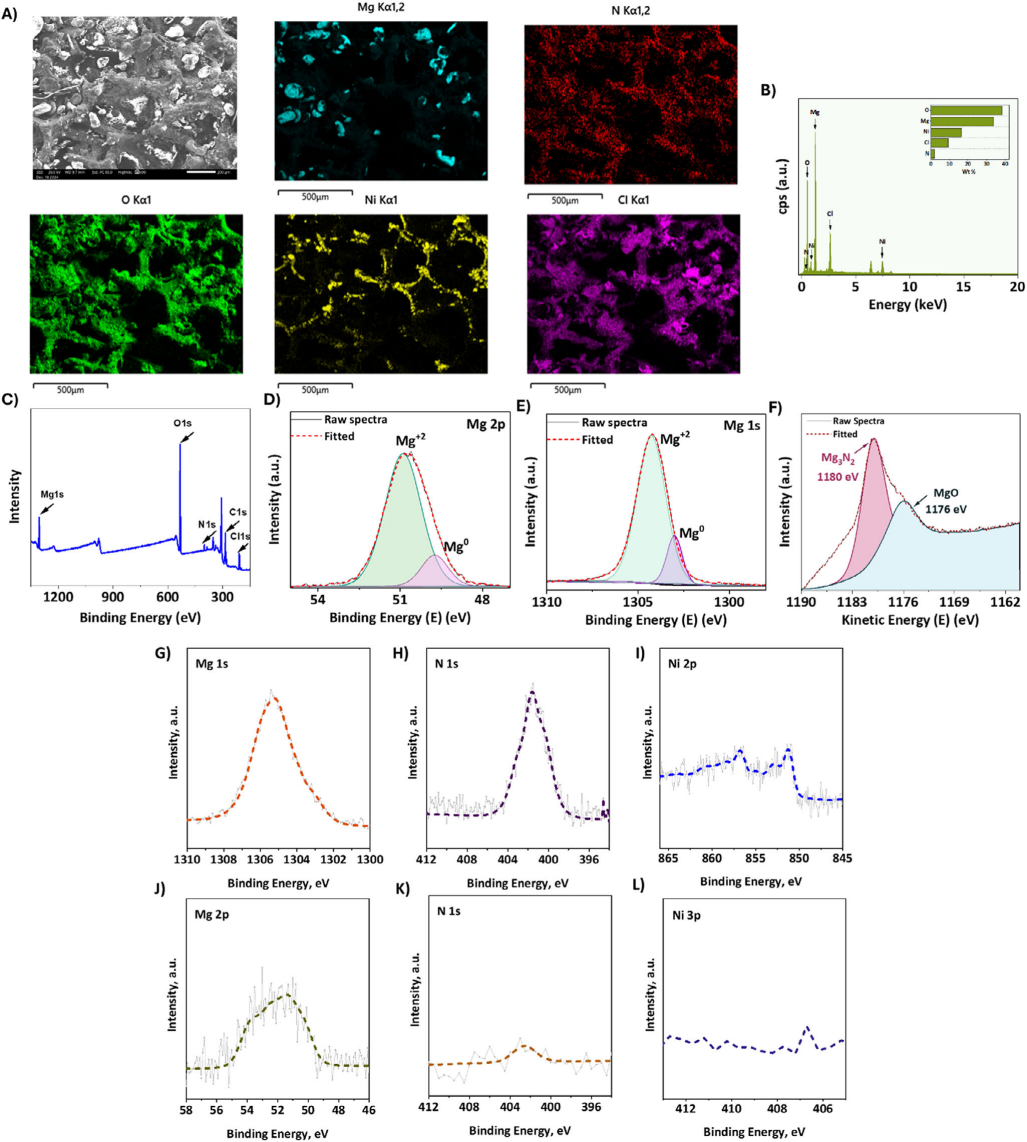
A) Scanning electron microscopy (SEM) images, energy dispersive X‐ray spectroscopy (EDS) spectra, and elemental mapping of Mg, O, Cl, Ni, and N on Ni foam post‐electrolysis cathode. B) EDS spectrum of post‐electrolysis electrode showing the presence of Mg and N. C) X‐ray photoelectron spectroscopy (XPS) Survey scan of the post‐reaction electrode showing the presence of Mg and N. D) High‐resolution XPS scan of the post‐electrolysis electrode confirming the presence of Mg^0^ (1s) E) High‐resolution XPS scan of the post‐electrolysis electrode confirming the presence of Mg^0^ (2p). F) High‐resolution Mg KLL XPS auger scan, which clearly shows the presence of Mg_3_N_2_. G, H, I, J, K, L) Depth‐profiling XPS spectra for Mg *1s*, Mg *2p*, N *1s*, Ni *2p*, and Ni *3p* of the post‐electrolysis catalyst. The top set was probed using tender X‐rays at 4000 eV (10 nm), while the bottom set of spectra was recorded using soft X‐rays at 900 eV (2 nm).

The post‐electrolysis catalyst was characterized using scanning electron microscopy with energy dispersive X‐ray spectroscopy (SEM‐EDS) and X‐ray photoelectron spectroscopy (XPS) on the post‐reaction sample, as illustrated in **Figure** **4**. SEM‐EDS analysis of the post‐electrolysis Ni foam revealed the presence of Mg and N on the electrode, as demonstrated in the EDS mapping shown in **Figure** 4A,B. In **Figure** 4C, a survey XPS spectrum of the post‐electrolysis electrode, confirms the presence of Mg 1s and N 1s, which strengthens the evidence of Mg nitride formation. **Figure** 4D,E shows high‐resolution XPS spectra for Mg⁰ *1s* and Mg⁰ *2p* core‐level peaks, confirming the presence of Mg⁰ and strengthening the evidence for metallic Mg deposition during the electrochemical process. To resolve the uncertainty in XPS analysis about whether nitrogen signals originated from nitride formation or the nitrogen in DMF, Auger Mg KLL XPS analysis was conducted to distinguish between the observed peaks. The Mg₃N₂ peak was identified at a kinetic energy of 1190 eV, consistent with the findings of Bouvier et al.^[^ ^35^ ^]^ This result provides definitive evidence for the presence of Mg₃N₂ on the electrode surface, as shown in **Figure** 4F. To definitively establish that the observed nitrogen signal originates from magnesium nitride (Mg₃N₂) rather than residual DMF, we performed comprehensive XPS characterization. First, to confirm that the nitrogen peak results from Mg₃N₂ formation rather than electrolyte's DMF, we analyzed the pre‐electrolyte through a control experiment. Ni foam was immersed in Mg(ClO₄)₂/DMF solution for 2 h and vacuum‐dried for 96 h, yielding an N *1s* peak at 399 eV (**Figure** S16A, Supporting Information) – markedly different from the characteristic Mg₃N₂ peak at 401 eV observed in post‐reaction electrodes, thereby excluding DMF as the nitrogen source. To further verify that the magnesium signal derives from Mg₃N₂ rather than surface MgO, we employed Auger XPS analysis of air‐exposed pure Mg powder (predominantly MgO). Auger XPS is capable of distinguishing between MgO and Mg₃N₂, allowing for precise identification of the magnesium species. The resulting spectrum (**Figure** S16B, Supporting Information) showed only the MgO peak at 1176 eV, while the post‐reaction electrode clearly displayed both Mg₃N₂ (1180 eV) and MgO peaks. Crucially, the post‐electrolysis spectrum revealed these distinct features alongside an MgO signal that precisely aligned with the reference Mg powder spectrum, providing unambiguous evidence for Mg₃N₂ formation. These systematic comparisons yield conclusive evidence that: (1) the nitrogen signature arises exclusively from electrochemically generated Mg₃N₂, not residual DMF, and (2) magnesium nitride (Mg₃N₂) and magnesium oxide (MgO) can be distinguished using Auger XPS analysis and the post‐reaction electrode contains Mg₃N₂. The collective XPS data thus provide robust support for our assignment of the nitrogen species to electrochemically synthesized magnesium nitride during nitrogen reduction. Moreover, a Ni foam (0.5 mm thickness) sample was carefully prepared because it was observed that it could easily release any residual DMF at high temperatures and didn't retain a wet appearance. Mg was coated onto the Ni foam, and N₂ was bubbled continuously to facilitate nitride formation. To protect the nitride layer from reacting with air, a thin layer of copper was deposited onto the Ni foam. The sample was then dried in a vacuum oven for 96 h to ensure all traces of DMF were removed. Finally, a depth profile analysis was carried out to examine the SEI layers beneath the copper coating. Depth‐profile XPS measurements were conducted to investigate the composition of the post‐electrolysis catalyst using synchrotron‐based XPS at the Advanced Light Source (ALS), specifically at beamlines 9.3.2 and 9.3.1. Tender X‐rays with a photon energy of 4000 eV provided information from a depth of ≈10 nm below the surface (**Figure** 4G–I), while soft X‐rays probed at a shallower depth of ≈2 nm (**Figure** 4J–L).

The successful electrodeposition of a relatively thick (>12 nm) Mg layer, along with the near‐complete coverage of the nickel foam substrate, is evidenced by the absence of a nickel signal at the top surface of the catalyst (2–4 nm, probed at 900 eV) and a weak nickel signal in the XPS depth‐profile at 10 nm (4000 eV).

The analysis of the Mg *1s* and *2p* core‐level spectra, combined with the N *1s* spectrum, revealed that surface Mg species are primarily nitrogen‐deficient, consisting of oxides, hydroxides, and carbonates.^[^ ^36^, ^37^ ^]^ However, probing deeper into the catalyst layer (10 nm) with tender X‐rays demonstrated the presence of nitrogen‐rich Mg nitride phases. These are represented by broad peaks at ≈1305 eV (Mg *1s*) and ≈400 eV (N *1s*), likely corresponding to Mg₃N₂ and possibly another nitride phase (e.g., MgN).^[^ ^38^ ^]^ The presence of nitride‐related peaks suggests surface interactions between the deposited Mg and nitrogen during ammonia synthesis.

To determine the physical characteristics of the SEI layer, we conducted the experiment using a polished planar metal electrode as the cathode. The cell setup remained the same, with the only difference being the replacement of the 3 mm thick Ni foam with the planar electrode. This facilitated faster drying of the solvent and provided improved imaging of the SEI structure. SEM images and EDS mapping, as shown in **Figure** S8 (Supporting Information), revealed the deposition of a thick SEI layer on the planar electrode. The mapping also showed a dense presence of Mg, further confirming that Mg deposition mediates the ammonia synthesis, supporting the direction of the proposed mechanism to a greater extent. These ex‐situ analysis findings collectively reinforce the conclusion that Mg was successfully deposited during electrolysis.

**Figure 5:**
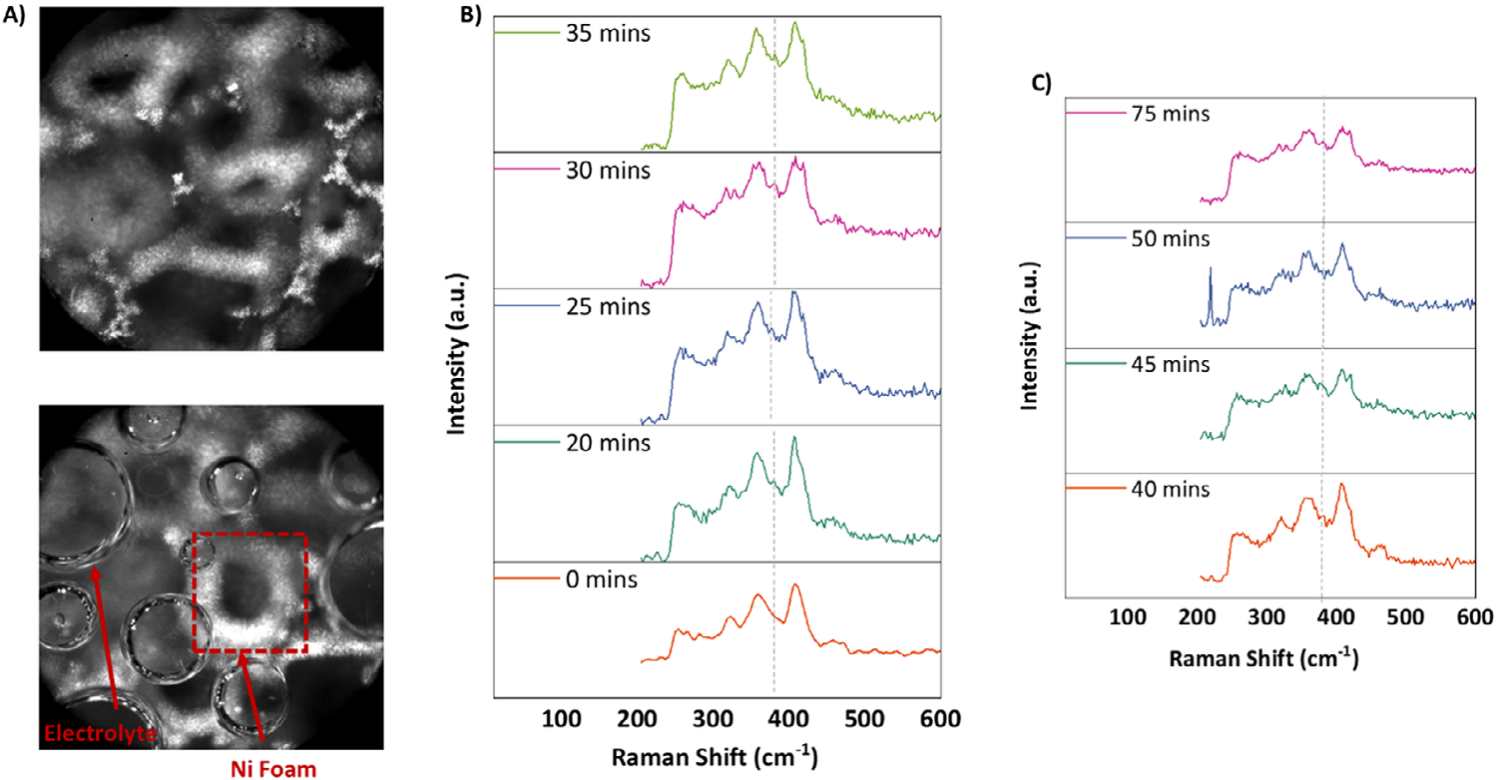
In‐situ Raman spectroscopy analysis of magnesium nitride (Mg₃N₂) formation and consumption during nitrogen reduction. A) Optical image of the cathode surface (Ni foam/electrolyte interface) during in‐situ Raman measurements. B) Time‐dependent Raman spectra (0 min) showing the progressive increase in the Mg₃N₂ peak intensity, indicating nitrogen activation. C) Spectra from 40 min onward reveal a decline in the Mg₃N₂ signal, corresponding to its consumption during ammonia production followed by its stabilization.

To probe the evolution of the solid‐electrolyte interphase during magnesium plating, we conducted in‐situ Raman spectroscopy to assess potential Mg₃N₂ formation. A custom electrochemical cell was employed, featuring a platinum counter electrode and a 3 mm‐thick nickel foam working electrode. To maintain a controlled atmosphere with minimal fluid disturbance, nitrogen gas was continuously purged through a needle inserted into the sparging tube, regulated by a mass flow controller (**Figure** **5**A). Raman spectra were acquired over 1 h at regular intervals, revealing a distinct peak at ≈379 cm⁻¹, corresponding to Mg₃N₂ (**Figure** 5B,C), in agreement with prior reports.^[^ ^1^ ^]^ Additional peaks correspond to the perchlorate electrolyte.^[^ ^2^ ^]^ The temporal evolution of this peak provides insight into the reaction dynamics: initially appearing as a weak shoulder, the signal intensifies gradually, indicating progressive magnesium deposition and subsequent nitride formation. However, toward the end of the reaction, the peak diminishes, suggesting rapid consumption of Mg₃N₂, likely due to further reaction with nitrogen to form ammonia and then its stabilization. These findings confirm magnesium plating on the Ni foam cathode and demonstrate the concomitant formation and reactivity of magnesium nitride within the SEI layer.

The research outlined in this letter provides an initial demonstration of Mg‐mediated NH_3_ synthesis, serving as proof of concept. We achieved a maximum NH_3_ FE of 25.28 ± 3.8% at a current density of −45 mA cm^−2^, which amounts to an NH_3_ current density of −11.3 ± 1.77 mA cm^−2^. While Li‐mediated NH₃ synthesis has made significant progress, the scarcity of Li and its highly negative plating potential (≈−3.04 V versus SHE) limit scalability and energy efficiency. Ca has been explored as an alternative, achieving NH₃ FE values around 50%, though with cell voltages exceeding 3 V, making it less practical. In contrast, this study presents Mg‐NRR process, where N₂ is activated on Mg to form nitride, followed by its protolysis to produce NH₃, coupled with the regeneration of Mg sites. This approach demonstrates the potential of Mg as a more sustainable and energy‐efficient mediator, paving the way for future research into Mg and other metals beyond Li for ammonia production. In this study, we validated the reduced plating potential for Mg compared to Li and Ca. Remarkably, using Mg‐NRR, we achieved ammonia synthesis at a working electrode potential as low as −3 V. Furthermore, we found that NH_3_ FE remains constant for various current densities. Lithium‐mediated systems have been optimized to achieve ≈100% Faradaic efficiency for ammonia synthesis since Fichter's work in the 1930s, whereas Mg‐mediated Mg‐NRR remains in its infancy—our work representing only the second functional demonstration. Despite its early stage, magnesium offers key advantages as an earth‐abundant metal: its higher reduction potential (.4 V versus SHE) enables 25% greater theoretical energy efficiency compared to Li (.0 V). Critically, this higher potential also reduces electrolyte decomposition. However, the process encounters several challenges that must be addressed for further development. One major obstacle is the limited solubility of Mg salts in the solvents explored in this study. Future investigations should focus on screening the solubility of different Mg salts in various aprotic solvents. This exploration would facilitate the testing of different salt and electrolyte configurations for Mg‐mediated NH_3_ synthesis, ultimately leading to an optimal setup to maximize NH_3_ FE and lower the total cell potential, thereby enhancing the overall energy efficiency of the process. Additionally, the role of the SEI in determining NH_3_ FE remains poorly understood. Overall, the findings presented here pave the way for future research on NH_3_ synthesis utilizing Mg and other earth‐abundant materials beyond Li as potential mediators.

## Conflict of Interest

A PCT application titled “Metal Nitride Mediated Ammonia Synthesis” has been filed.

## Author Contributions

Ishita Goyal conceived the idea, planned and performed all the experiments, and wrote the manuscript. Vamsi Vikram Gande assisted with the experiments and co‐wrote the manuscript with Ishita Goyal. Rajan R. Bhawnanicontributed to the XPS characterization. Rebecca Hamlyn and Ahmed A. Farghaly assisted with the XPS depth profiling experiments. Meenesh R. Singh conceived the idea, planned and supervised the project, and co‐wrote the manuscript.

## Supporting information



Supporting Information

## Data Availability

The data that support the findings of this study are available in the supplementary material of this article.
