# Supplementary material for: Magnesium‐Mediated Electrochemical Synthesis of Ammonia
Source: Adv Sci (Weinh). 2025 May 15;12(28):2504882. doi: 10.1002/advs.202504882 (PMC12302589; doi:10.1002/advs.202504882)
Supplement: Supplementary file 1 — Supporting Information [file ADVS-12-2504882-s001.pdf]

ADVANCED SCIENCE
Open Access

Supporting Information

for *Adv. Sci.*, DOI 10.1002/advs.202504882

Magnesium-Mediated Electrochemical Synthesis of Ammonia

*Ishita Goyal, Vamsi V. Gande, Rajan R. Bhawnani, Rebecca Hamlyn, Ahmed A. Farghaly and Meenesh R. Singh**

WILEY
ADVANCED

Supplementary Information for

Magnesium-Mediated Electrochemical Synthesis of Ammonia

Ishita Goyal[a], Vamsi V Gande[a,b], Rajan R. Bhawnani[a], Rebecca Hamlyn[c], Ahmed A. Farghaly[d,e], and Meenesh R. Singh[a]*

[a]Department of Chemical Engineering, University of Illinois Chicago, Chicago, Illinois, 60607 United States

[b]Department of Chemical Engineering, Indian Institute of Technology Hyderabad, 502285, India

[c]Chemical Sciences Division, Lawrence Berkeley National Laboratory, Berkeley, California 94720, United States

[d]Chemical Sciences and Engineering Division, Argonne National Laboratory, Lemont, Illinois, 60439 United States

[e]Pritzker School of Molecular Engineering, The University of Chicago, Chicago, Illinois, 60637 United States

***Corresponding Author:**

Prof. Meenesh R. Singh
Email: mrsingh@uic.edu

This PDF file includes:

Supporting text

Figures S1 to S16

Tables S1 to S4

Methods and Materials

Electrochemical Experiments

The magnesium mediated electrochemical synthesis of ammonia was conducted using a specialized 2-electrode setup, comprising a single-compartment glass cell within a custom-designed autoclave placed in an explosion-proof enclosure. The electrolyte utilized was a solution of 0.5 M magnesium perchlorate dissolved in dimethyl formamide (DMF), with an addition of 0.065 M ethanol (EtOH), pre-saturated with nitrogen (N2) for 10 minutes at a flow rate of 200 standard cubic centimeters per minute (sccm). Nickel (Ni) foam was employed as the cathode, with fresh Ni foam used for each run. Before the reaction, the Ni foam was cleansed with ethanol to eliminate water contamination, followed by drying in an oven at 80°C for 20 minutes. A planar platinum (Pt) anode was utilized, cleaned after each reaction by sonication in acetone and ethanol, followed by oven drying at 80°C for 20 minutes. The Ni foam and Pt anode were electrically connected using copper (Cu) wire and Cu tape. The glass cell and magnetic stirring bar were dried at 80°C for 1 hour before assembly. The distance between the anode and cathode was approximately 1 cm, with a cathode surface area of 4 cm^2 facing the anode. Before electrochemical experiments, the autoclave was purged with N2 from a gas cylinder 10 times to remove oxygen and other contaminants and then pressurized to a desired pressure of 6 bars. Electrochemical experiments were carried out using a potentiostat (Biologic SP 300) with stirring at 700 rpm. The stirring rate was adjusted if the open circuit potential deviated from the desired limit, as demonstrated for one experiment in Figure S15. Pulsed Chronopotentiometry was conducted following the switching current strategy proposed by Anderson et al., with a current density of -5 mA/cm^2 for 1 minutes, followed by 0 mA/cm^2 for 1-6 minutes, depending on the need to change, stabilize, or adjust the working electrode potential. During the working cycle various current densities ranging from -5 mA/cm^2 up to -45 mA/cm^2 were tested. All experiments were conducted at room temperature.

The CV experiments were conducted using the same reactor set up. 3 mm Ni foam electrode served as the working electrode and a platinum slab as the counter electrode. Same protocol as mentioned above was followed to pressurize the reactor with 6 bar of nitrogen. The potential was scanned between -4 V and +4 V vs Ag/AgCl at a scan rate of 10 mV/s. Data acquisition was performed using EC-Lab and all measurements were carried out at room temperature. The CV data was analyzed to identify cathodic and anodic peaks corresponding to magnesium deposition and stripping, and the stability of the electrolyte was confirmed during repeated cycles.

Experimental Procedure for Propylene Carbonate System

Experiments using propylene carbonate were conducted by dissolving 1M $Mg(ClO_4)_2$ in propylene carbonate, along with 0.065M ethanol (EtOH). The solution was thoroughly mixed using a vortex mixer to ensure complete dissolution of the salt. Once fully dissolved, the solution was purged with nitrogen (N_2) at a flow rate of 100 sccm for 10 minutes to remove any dissolved oxygen.

The experimental protocol followed for this system was similar to that used with DMF. Pulsed Chronopotentiometry was performed with alternating working and resting periods of 1 minute each, using a current density of 2 mA/cm^2 and 30 mA/cm^2. A higher current density of 50 mA/cm^2 was also tested; however, the voltage required for this condition was excessively high, causing an overload in the potentiostat. The post-electrolysis solutions were analyzed using NMR, and no ammonia peaks were detected.

Experimental Procedure for preparation of Ni foam sample for depth profiling analysis

The Ni foam sample for depth profiling XPS analysis was prepared using a 0.5 M $Mg(ClO_4)_2$ solution in 30 mL of dimethylformamide (DMF) without ethanol. The experiment was conducted in an open 50 mL beaker, with the 0.5 mm Ni foam serving as the cathode and a platinum (Pt) slab as the anode. Chronoamperometry was performed by applying a constant voltage of -4 V to the working electrode for 1 hour, with nitrogen gas (N_2) continuously bubbled through the electrolyte at a flow rate of 50 sccm. After the reaction, N_2 bubbling

was maintained for an additional 15 minutes without any applied potential. Subsequently, 0.1 M copper nitrate hydrate was added to the electrolyte solution, and chronoamperometry was conducted at -1 V for 30 minutes, while N_2 bubbling continued. This process resulted in the deposition of copper onto the Ni foam, which was then removed and dried for 96 hours in vacuum oven at 135°C and prepared for XPS depth profiling.

Colorimetric quantification of products

NH_3 was quantified by the Indophenol blue method using a standard additions method to account for the changes in appearance and properties of the electrolyte solution after the electrochemical reaction. It was quantified in both the electrode deposits and the electrolyte. After each experiment, the electrode was immersed in the post-reaction samples and sonicated for 30 minutes to ensure that all the ammonia present in the electrode deposits was fully mixed with the electrolyte. In this method, post electrolyte sample is diluted 100-400 folds with 0.1M H_2SO_4 to bring the concentration to the detectable limits. After dilution the ammonia present will be in the form of ammonium ion. Subsequently, the solution is centrifuged twice for 15 minutes each to obtain a clear solution. Calibration solutions are prepared using 0.01M NH_4Cl as the stock solution and further diluting it to make 100-500uM NH_4Cl calibration solutions in H_2SO_4. The blank solution for prepared by adding, 500µL of H_2SO_4, 500µL of Phenol nitroprusside and 500µL of alkaline sodium hypochlorite. 6 sample vials were each filled with 400µL aliquots of the centrifugated diluted post electrolyte solution and 100 µL of internal aqueous standard solutions (0.1M H_2SO_4, 100uM-500uM NH_4Cl in 0.1M H_2SO_4) along with 500 µL of Phenol nitroprusside followed by 500 µL of alkaline sodium hypochlorite. The mixtures were incubated in dark for 30 minutes at ambient temperature. The sample changes color from colorless to blue. The sample was scanned for absorbance as a function of wavelengths from 400 to 800 nm using a visible spectrometer (*Genesys 30 Visible Spectrometer*). The absorbance vs wavelength plot for one of the post electrolyte solution is shown in Figure S2B. The maximum absorbance was observed at 632 nm and hence 632 nm was chosen to measure absorbances to quantify NH_3. Absorbances of all the six solutions at 632 nm is plotted linearly against concentration as shown in Figure S2A. The ratio of the intercept and the slope is the moles of ammonia in the test sample. After calculating the moles, concentration and partial current density is determined.

Table S1: Details of the chemicals used for the current study.

S. No.	Chemicals	Purity (%)	Catalog Number	Manufacturer
1	Magnesium perchlorate	>97%	401420-100G	Sigma Aldrich
2	Ethyl Alcohol	99.5%	459836-4X2L	Sigma Aldrich
3	Phenol Nitroprusside	NA	P6994	Sigma Aldrich
4	Alkaline Sodium Hypochlorite	NA	A1727-120ML	Sigma Aldrich
5	Sulfuric Acid	0.1M	68279-1L	Sigma Aldrich
6	Acetone	99.5%	179124-4X4L-PB	Sigma Aldrich
7	Dimethyl sulfoxide	>99.9%	276855-1L	Sigma Aldrich
8	Ammonium Chloride	99.5%	A9434-500G	Sigma Aldrich

9	Dimethoxy ethane	99.9%	259527-1L	Sigma Aldrich
10	Dimethyl formamide	99.8%	227056-1L	Sigma Aldrich
11	Nitrogen gas	99.999%	NI 5.0UH-T	Linde
12	Argon Gas	99.999%	AR 5.0UH-T	Linde
13	Propylene carbonate	99.7%	310328	Sigma Aldrich
14	Dimethyl sulfoxide-d6	99.9%,	1034240100	Sigma-Aldrich
15	^{15}N Ammonium Chloride	99.9%	39466-62-1	Sigma-Aldrich
16	$^{15}N_2$ gas	98%	29817-79-6	Cambridge Isotope laboratories
17	Copper Nitrate hydrate	99%	229636-100G	Sigma-Aldrich
18	Magnesium	99%	13112-100G	Sigma-Aldrich

Table S2: Details of the materials used for the current study.

S. No.	Materials	Manufacturer
1	Nickel Foam	Fuwarmth
2	Platinum Electrode	ACI Alloys
3	Autoclave	Parr Instruments

Table S3: Ammonia Yield with different total current densities

Total Current Applied mA/cm^2	Ammonia FE %	Yield using NMR Mol/cm^{2}s	Yield using UV-Vis Mol/cm^{2}s
-5	28.72	2.5E-9	2.3E-9
-10	28.03	4.85E-9	4.78E-9
-15	27.15	7.03E-9	7.1E-9
-30	26.79	1.4E-8	1.54E-8
-45	25.28	1.98E-8	2.01E-8

Table S4: Experimental conditions for various metal mediated ammonia synthesis approaches.

Metal	Current applied mA/cm^2	Faradaic Efficiency %	Cell Voltage V	Electrolyte composition	N_2 pressure Bar
Li-NRR	-5	18.7	~-4.5	2M $LiClO_4$ + 0.065M EtOH in THF	6
Li-NRR	-15	21.4	~-4.75	2M $LiClO_4$ + 0.065M EtOH in THF	6
Li-NRR	-30	17.3	~-5.5	2M $LiClO_4$ + 0.065M EtOH in THF	6

Li-NRR	-45	19.7	~-6.3	2M $LiClO_4$ + 0.065M EtOH in THF	6
Ca-NRR	-5	33.6	~-3.32	0.5M $Ca(ClO_4)_2$ + 0.065M EtOH in DME	6
Ca-NRR	-15	49.3	~-4.65	0.5M $Ca(ClO_4)_2$ + 0.065M EtOH in DME	6
Ca-NRR	-30	27.2	~-6.89	0.5M $Ca(ClO_4)_2$ + 0.065M EtOH in DME	6
Ca-NRR	-45	26.8	~-8.35	0.5M $Ca(ClO_4)_2$ + 0.065M EtOH in DME	6
Mg-NRR	-5	28.7	~-3.01	0.5M $Mg(ClO_4)_2$ + 0.065M EtOH in DMF	6
Mg-NRR	-15	28.0	~-3.6	0.5M $Mg(ClO_4)_2$ + 0.065M EtOH in DMF	6
Mg-NRR	-30	27.2	~-3.75	0.5M $Mg(ClO_4)_2$ + 0.065M EtOH in DMF	6
Mg-NRR	-45	26.8	~-5.5	0.5M $Mg(ClO_4)_2$ + 0.065M EtOH in DMF	6

Characterisation of the post electrolyte samples and the post electrolysis electrode

Nuclear magnetic Resonance (NMR) for quantification of $^{14}NH_3$ and $^{15}NH_3$ in post electrolyte sample

Ammonia was quantified in both the electrode deposits and the electrolyte. After each experiment, the electrode was immersed in the post-reaction samples and sonicated for 30 minutes to ensure that all the ammonia present in the electrode deposits was fully mixed with the electrolyte. NMR analyses were conducted using a Bruker Avance III 600 MHz system with dimethylsulfoxide-d6 (DMSO-d6) as the deuterated solvent. To prepare the sample, 1 mL of post-electrolyte was mixed with 200 µL of 98% H_2SO_4 and 800 µL of water to convert all NH_3 into ammonium sulfate. The mixture was thoroughly homogenized, and 570 µL of the resulting solution was transferred to an NMR tube, along with 30 µL of DMSO-d6, for analysis. The measurements were performed on the Bruker Avance III 600 MHz NMR instrument, with data acquisition consisting of 16 accumulated scans. To suppress water resonance, excitation sculpting was applied using a 3-ms 180° shaped pulse centered at 4.612 ppm. One-dimensional experiments were optimized to effectively suppress multiple solvent peaks, employing the perfect-echo variant to minimize J-modulation for 600 MHz samples. A total of 1,024 transient scans were recorded with an interscan delay of 1 second. Each free induction decay (FID) was acquired with 64,000 complex points over an acquisition time of 3.4 seconds. The processed spectra were zero-filled to 64,000 real points, and an exponential apodization function with a line broadening factor (lb) of 0.3 Hz was applied before Fourier transformation. Data analysis was performed using MNova software, with the signal-to-noise ratio evaluated for both calibration and product samples.

Scanning Electron Microscopy (SEM) and Energy Dispersive Spectroscopy (EDS) Characterization of Post-Electrolysis Materials

Scanning Electron Microscopy (SEM) was conducted using a Hitachi SU8030 microscope equipped with a secondary electron detector (SED). Following the reaction, the electrode was dried in a vacuum oven at 100°C for 96 hours. Before imaging, the samples were mounted on aluminum stubs with carbon tape to ensure proper adhesion. SEM imaging was carried out at an accelerating voltage of 20 kV with a working distance of 12 mm. Spectral data were collected with an energy resolution of 2048 channels and an energy dispersion of 0.01 keV/channel over a live time of 29.88 seconds and a real-time of 32.79 seconds. The EDS detector identified elements based on their characteristic X-ray peaks, including nitrogen (N), oxygen (O), chlorine (Cl), nickel (Ni), and Magnesium (Mg). Elemental quantification was performed using standardless calibration, with matrix effects and detector efficiency taken into account. The spatial distribution of elements was analyzed via mapping mode, summing the spectra over the region of interest for higher signal intensity.

SEM analysis of Planar Electrode

A planar Fe electrode was used as the working electrode. After the reaction, the electrode was dried in a vacuum oven at 100°C for 96 hours. Prior to imaging, the samples were mounted on aluminum stubs using carbon tape to ensure proper adhesion. SEM imaging was performed with the microscope operating at an accelerating voltage of 15 kV and a working distance of 4 mm.

X-ray Photoelectron Spectroscopy (XPS) Characterization of Post-Electrolysis electrode

XPS was performed on a ThermoFisher NEXSA-G2 to analyze the surface of the deposition on the Ni electrode. The X-ray source type was monochromated Al K-Alpha. The survey scan was conducted between 0 and 1000 eV with a resolution of 1eV. The survey scan was averaged over 3 sweeps to minimize the noise. Following the survey scans, the elemental scan was conducted between 55 and 47 eV to identify the Mg 2p and 1311 and 1296 eV for Mg 1s peaks with a resolution of 0.1eV. To minimize the noise, the high resolution elemental scan for Mg was averaged over 10 sweeps.

XPS confirms the presence of Mg on the Ni substrate. Since XPS is a surface analysis technique, negligible signal from Ni was obtained, since the beam was focused on the deposited material. However, when the beam is focused on bare Ni foam, the Ni peaks were clearly observed.

A high amount of carbon observed in both cases suggests that the organic electrolyte was decomposed and was deposited on the Ni foam.

Depth-profiling XPS characterization was performed with soft (< 2 keV) and tender (2-6 keV) X-ray beamlines 9.3.2 and 9.3.1 at the Advanced Light Source at Lawrence Berkeley National Laboratory. Spectra were analyzed with CasaXPS software, with an energy calibration to adventitious carbon at 284.5 eV.

In-Situ Raman Spectroscopy

The Raman spectroscopy measurements were performed using a Wasatch 785 nm Raman system equipped with a 785 nm excitation laser, along with a Blaze Metrics optical imaging probe incorporating a 532 nm laser. The spectra were collected using an integration time of 20,000 ms (actual integration time of 3,222 ms) with the laser power set to 100%, a gain of 1.9, and zero offset. The system temperature was maintained at 5.06°C while the laser temperature was kept at -105.01°C, with a 5-second warm-up delay implemented prior to data acquisition. For the in situ electrochemical Raman experiments, a custom-designed electrochemical cell was employed featuring a Pt slab counter electrode, and a 1 cm^2 Ni foam working electrode. Spectra were acquired with exposure times ranging from 2 to 10 seconds to ensure optimal signal-to-noise ratios. The electrochemical measurements involved constant potential electrolysis at -50 mA/cm^2 for 75 minutes, with Raman spectra recorded at regular intervals throughout the experiment.

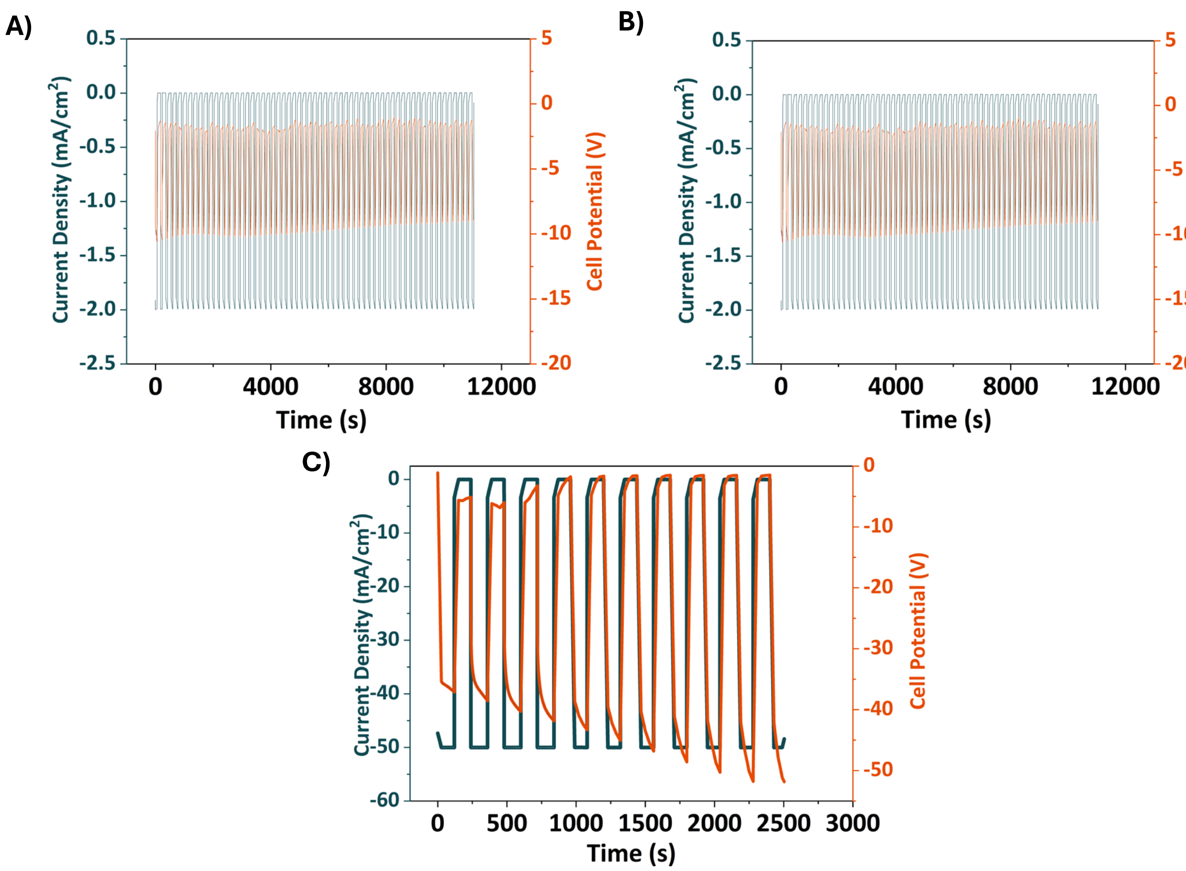

Figure S1: Pulsed Chronopotentiometry data for the propylene carbonate system: (A) Pulsed Chronopotentiometry profile at a current density of -2 mA/cm² with alternating 1-minute working and 1-minute resting periods. (B) Pulsed Chronopotentiometry profile at a current density of -30 mA/cm². (C) Pulsed Chronopotentiometry profile at a current density of -50 mA/cm², which was discontinued due to voltage overload in the potentiostat.

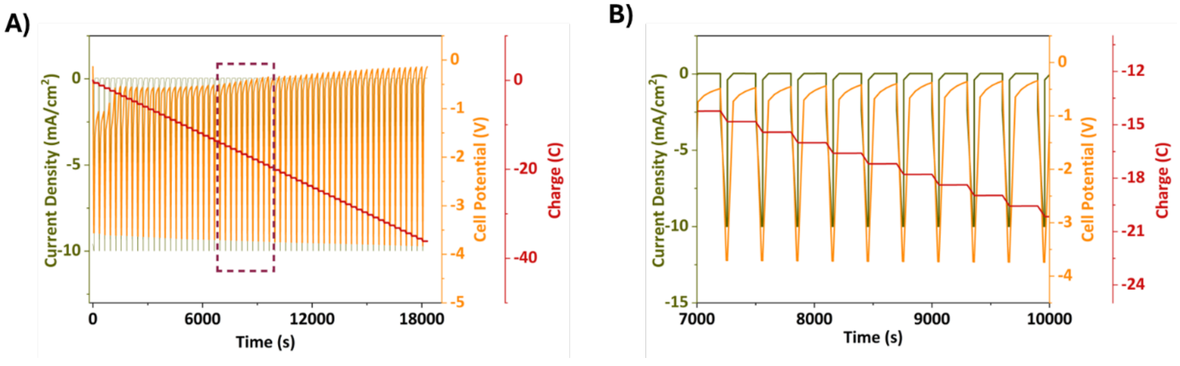

Figure S2: Representative Pulsed Chronopotentiometry A) Applied current density, total cell potential and charge as a function of time. Current density was switched between -10 mA/cm^2 and 0 mA/cm^2, the total charge passed was ~110 C. B) Zoomed version clearly denoting the applied current density, cell potential, and charge.

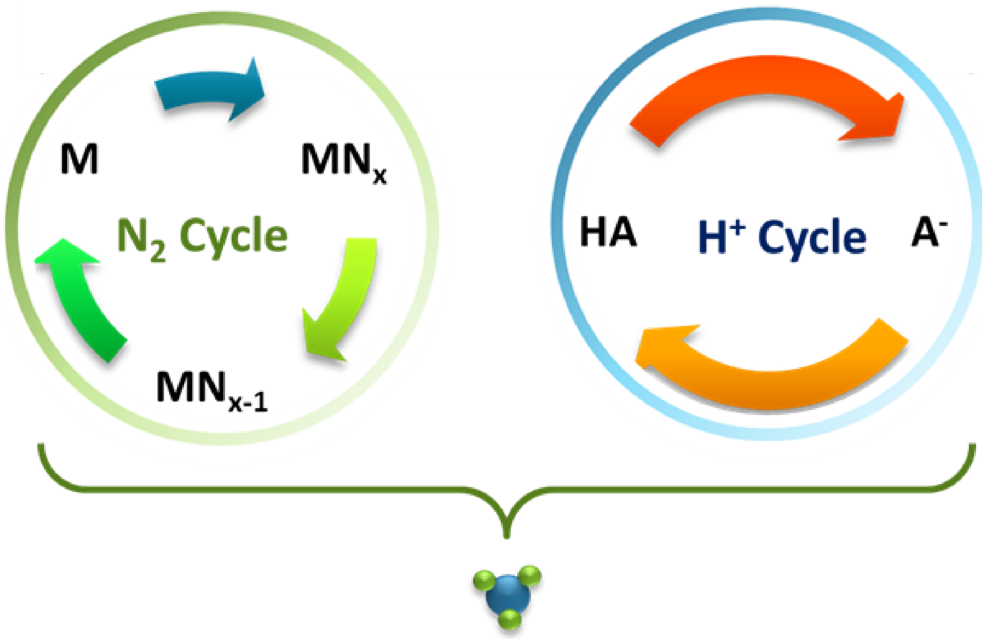

Figure *S*3: Nitridation and protonation cycles and their interdependence on each other.

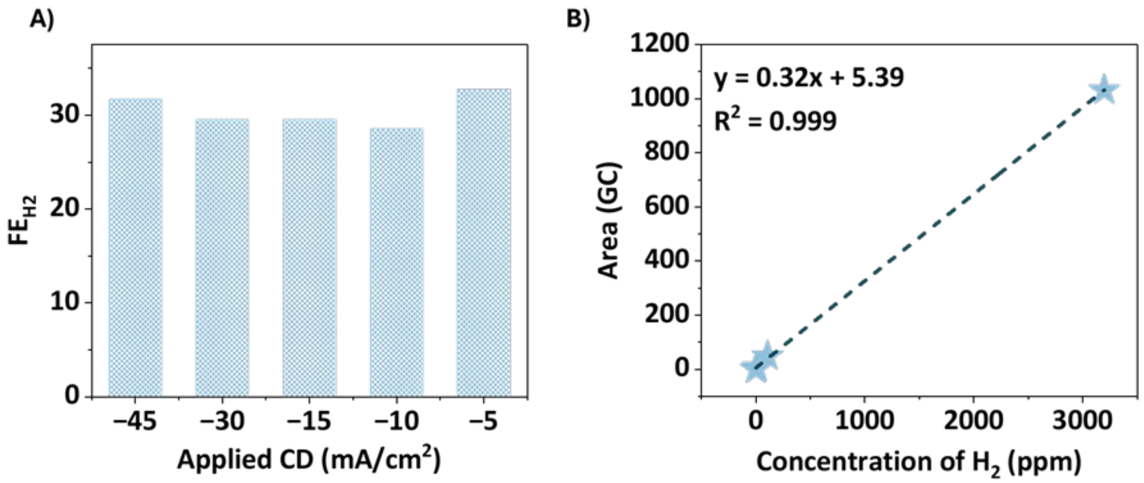

Figure S4: Faradaic efficiency of H_2 as a function of applied current density, ranging from -5 mA/cm² to -45 mA/cm², measured under 6 bar N_2 pressure. (B) Calibration curve for H_2 quantification using gas chromatography, constructed by plotting peak area versus known H_2 concentrations.

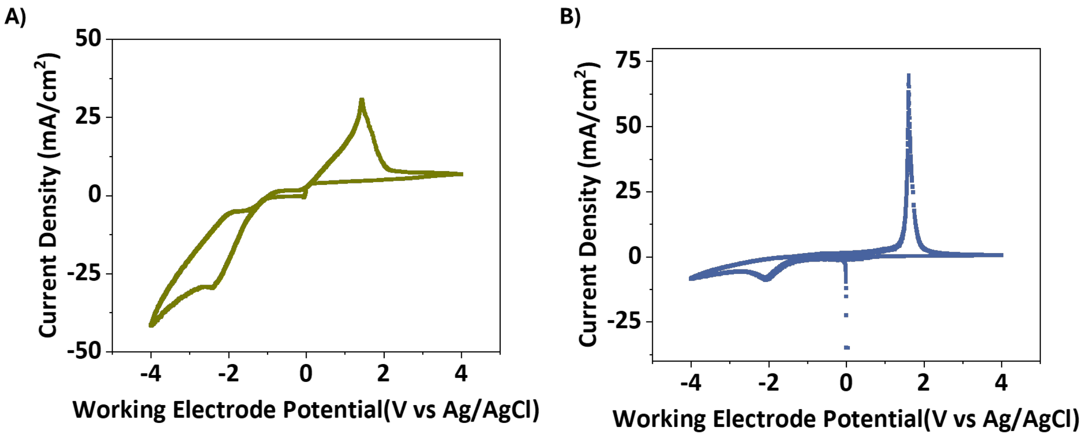

Figure S5: A)Cyclic voltammetry of 0.5M $Mg(ClO_4)_2$ in DMF with 0.065M ethanol at room temperature. The cathodic sweep indicates magnesium deposition at approximately -2.45 V vs. Ag/AgCl, and the anodic sweep shows stripping at 1.45 V vs. Ag/AgCl. B) Cyclic voltammetry of 0.5M $Mg(ClO_4)_2$ in DMF without ethanol at room temperature.

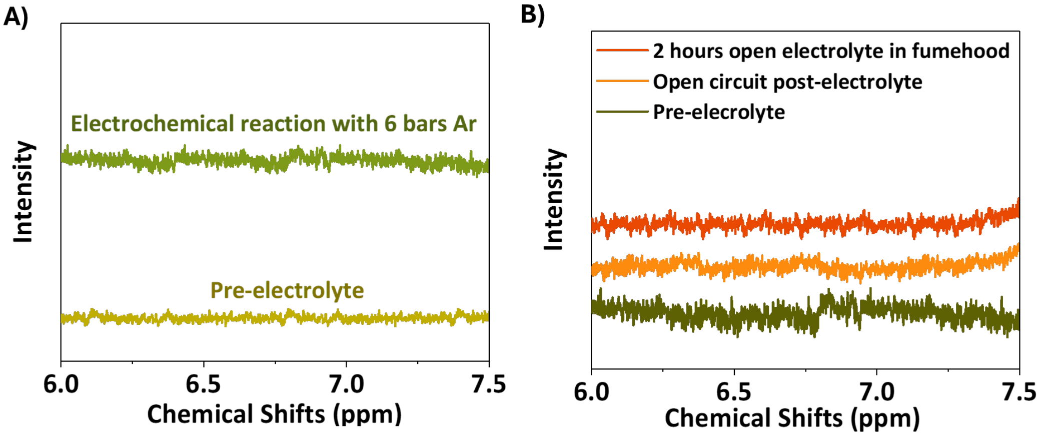

Figure S6: A) Post and pre electrolyte NMR spectra for control experiment under 6 bar Ar B) NMR spectra after keeping freshly prepared electrolyte open in the fume hood for 2 hours NMR, post and pre electrolyte spectra for open circuit control experiment.

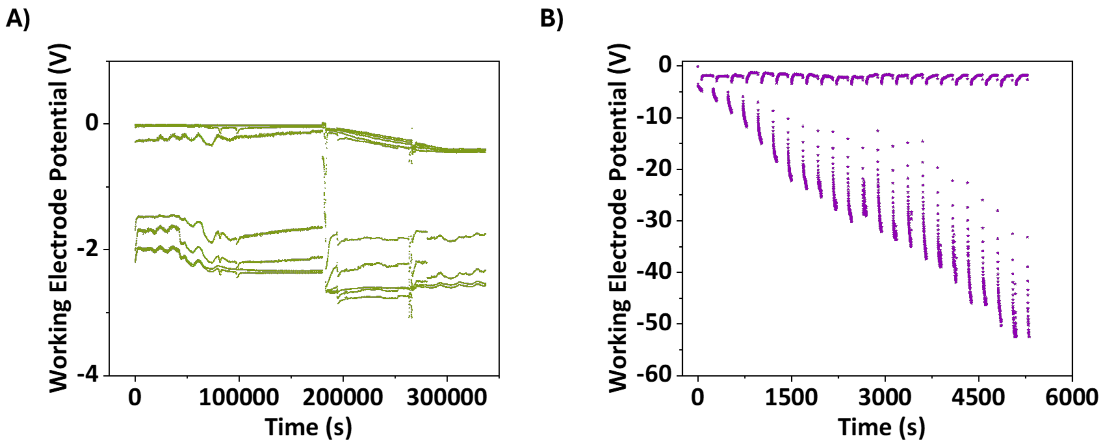

Figure S7: A) Long-term performance of Mg-NRR evaluated through a 94-hour stability experiment with 47 hours of cumulative working time. The cell was operated under a Pulsed Chronopotentiometry mode, alternating between a working current density of -5 mA/cm² for 1 minute and a resting current density of 0 mA/cm² for 1 minute. The cathode comprised a 3 mm Ni foam, while a Pt slab was used as the anode. The electrolyte consisted of 0.5 M magnesium perchlorate and 0.065 M ethanol dissolved in DMF. B) Pulsed Chronopotentiometry curve for magnesium-mediated ammonia synthesis at 6 bar N_2 pressure using a planar Ni metal slab as the working electrode. The system operated with a current density of -5 mA/cm² for 1 minute, followed by a 3-minute resting period at 0 mA/cm². Unlike Ni foam, the setup exhibited poor stability, with the potential increasing significantly after each cycle, eventually reaching the potentiostat limit of -50V. Electrolyte: 0.5 M $Mg(ClO_4)_2$ and 0.065 M EtOH in DMF; Anode: Pt slab.

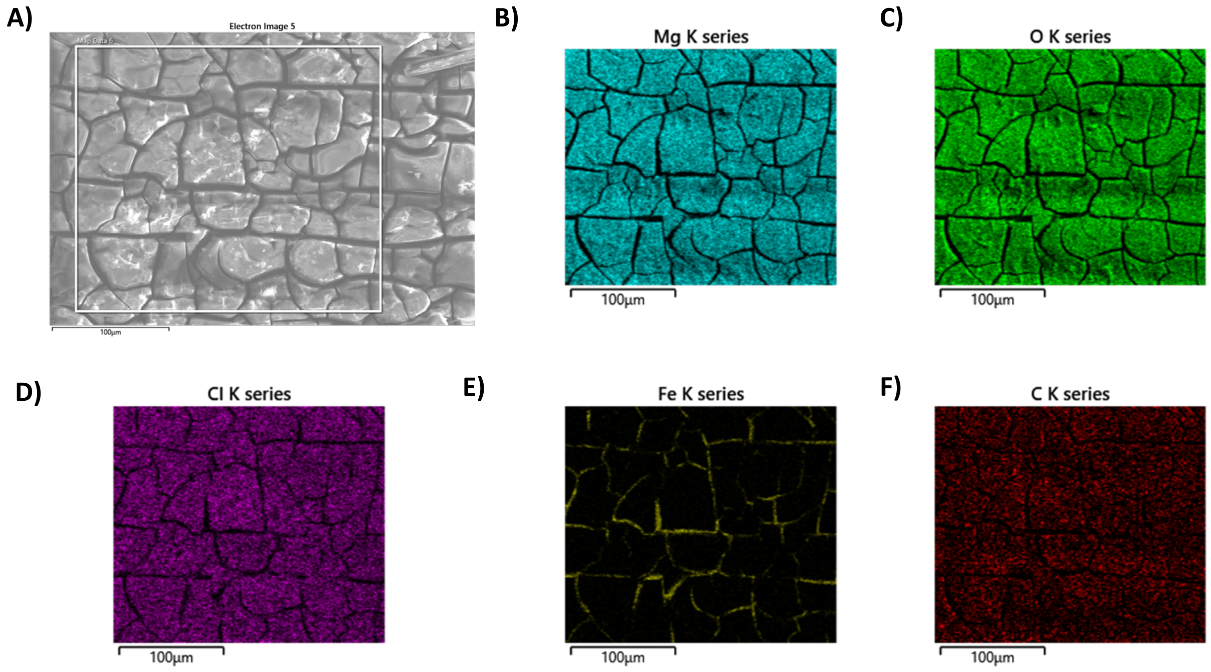

Figure S8: Scanning electron microscopy (SEM) images, energy dispersive X-ray spectroscopy (EDS) spectra and elemental mapping of Mg, O, C, Fe and Cl.

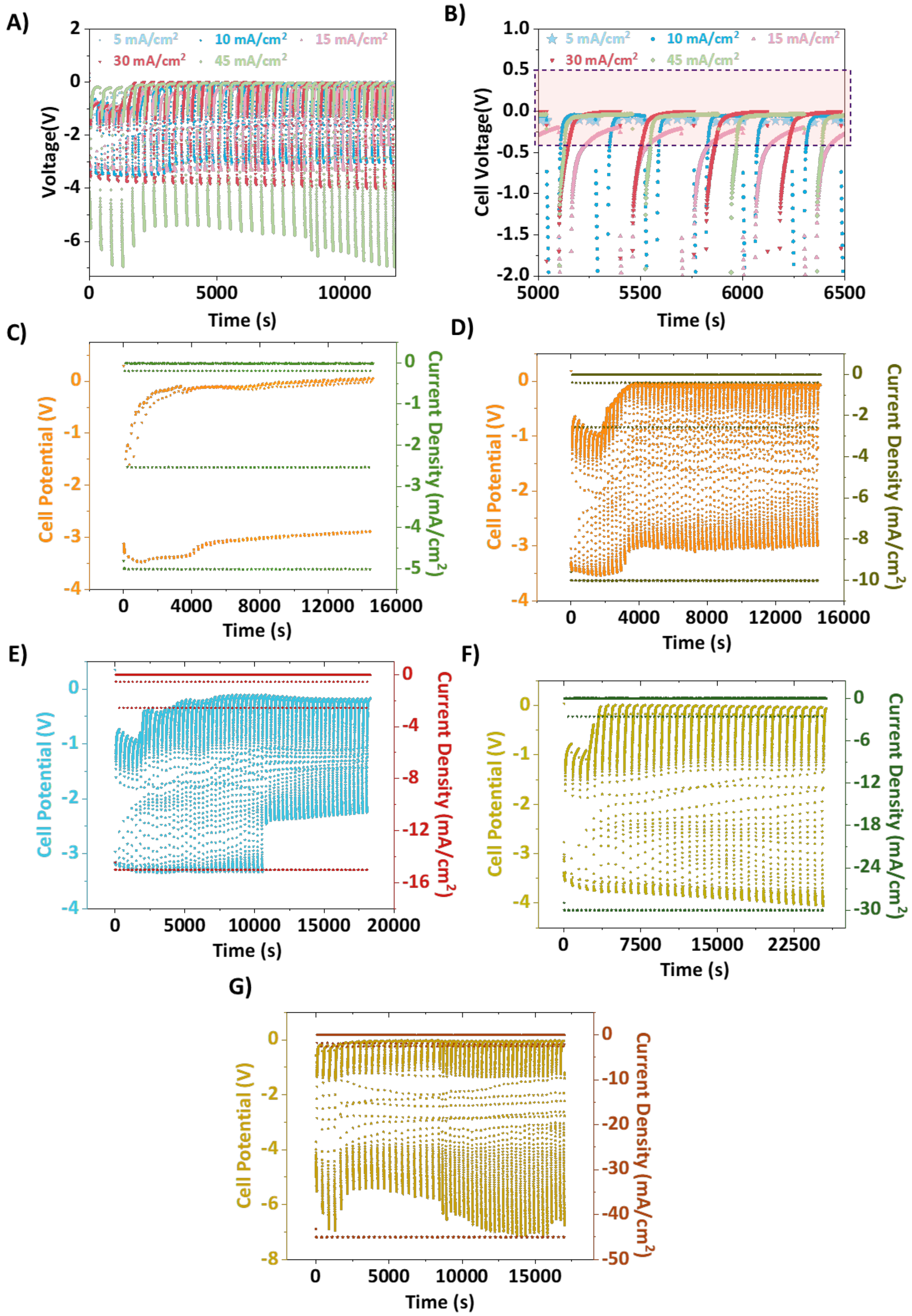

Figure S9: A) Cell potential vs. time at all current densities from 5 mA/cm² to 30 mA/cm² (working phase) and 0 mA/cm² (resting phase) B) Visual representation of different resting times (0 mA/cm²) for different working current densities (Operating conditions: Electrolyte – 0.5M $Mg(ClO4)_2$ + 0.065M EtOH in

dimethylformamide; 6 bar N2 pressure, Ni foam cathode and Pt anode). C) Pulsed Chronopotentiometry profile at a current density of -5 mA/cm² with alternating 1-minute working and 1-minute resting periods. (D) Pulsed Chronopotentiometry profile at a current density of -10 mA/cm² with alternating 1-minute working and 1-minute resting periods. (E) Pulsed Chronopotentiometry profile at a current density of -15 mA/cm² with alternating 1-minute working and 3-minutes resting periods. F) Pulsed Chronopotentiometry profile at a current density of -30 mA/cm² with alternating 1-minute working and 4-minutes resting periods. G) Pulsed Chronopotentiometry profile at a current density of -45 mA/cm² with alternating 1-minute working and 6-minutes resting periods.

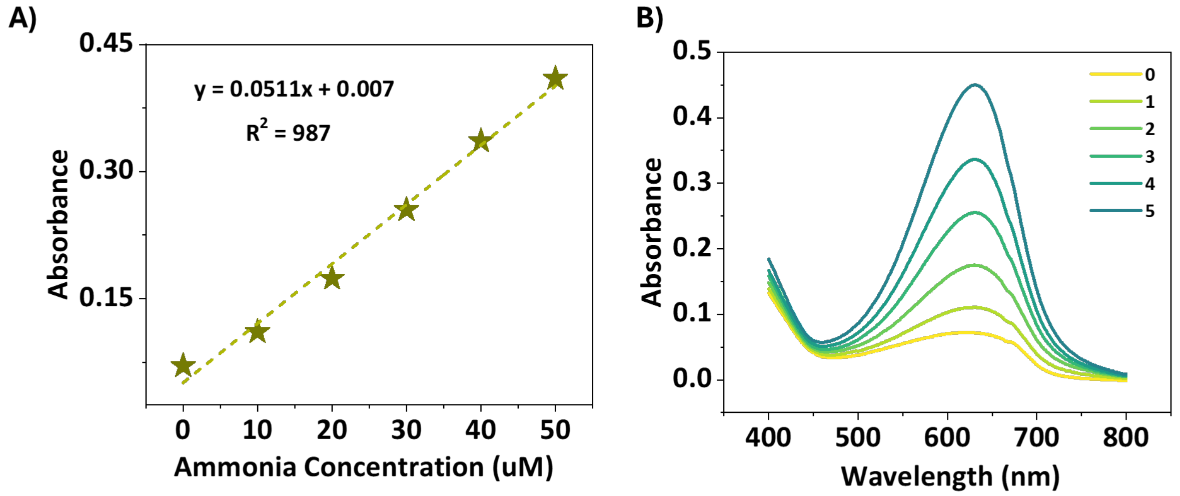

Figure S10: Calibration Graphs A) Absorbances as a function of NH_3 concentrations at 632 nm B) Absorbance scan as a function of wavelength.

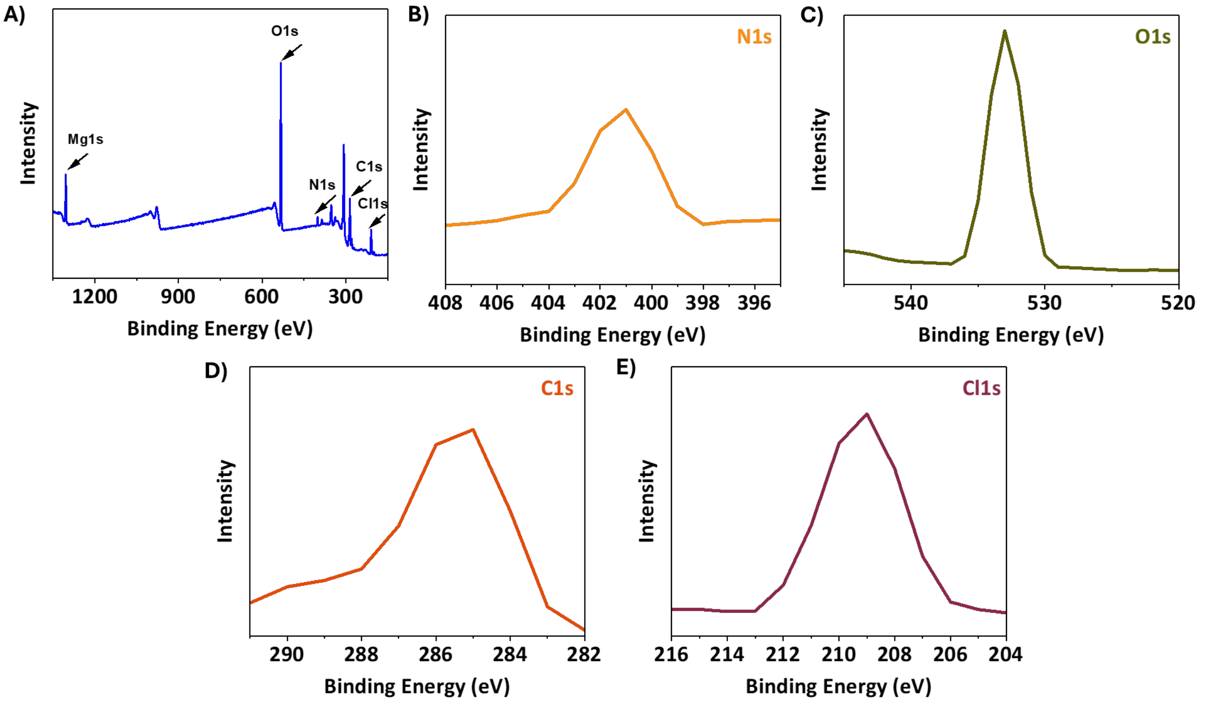

Figure S11: A) XPS Survey scan of the post reaction electrode. B) High resolution XPS scan of the post-electrolysis electrode indicating the presence of N. C) High resolution XPS scan of the post-electrolysis electrode indicating the presence of O. D) High resolution XPS scan of the post-electrolysis electrode indicating the presence of C. E) High resolution XPS scan of the post-electrolysis electrode indicating the presence of Cl.

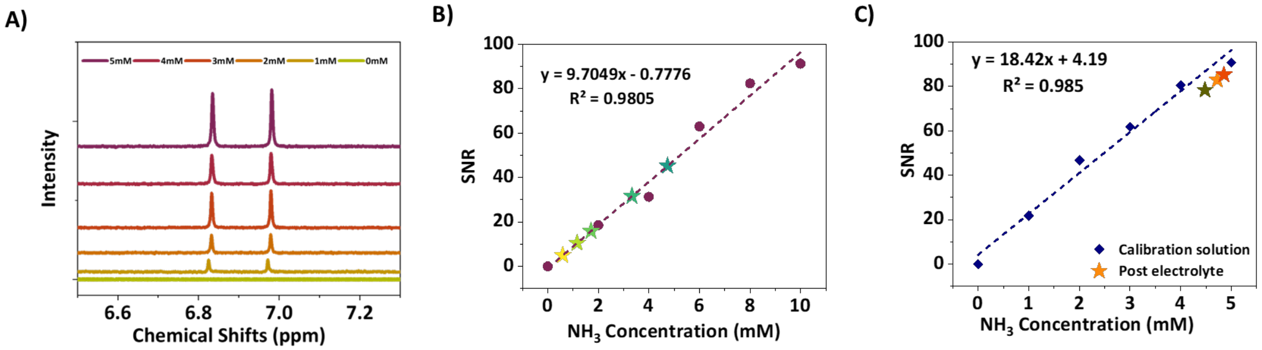

Figure S12: A) ^{1}H NMR spectra for isotope labelled standard ammonia solutions from 0mM to 5mM. B**)** Calibration curve generated from standard ammonia solutions (represented by dot symbols) prepared at known concentrations. The post-reaction product solutions (denoted by star symbols) are overlaid onto the calibration plot, with matching colors from Figure 5A to indicate which spectra correspond to which current densities. C) Calibration curve generated from standard isotope labelled $^{15}NH_3$ ammonia solutions (represented by blue diamond symbols) prepared at known concentrations. The isotope labelled post-reaction product solutions (denoted by star symbol) are overlaid onto the calibration plot, with matching color from Figure 5C.

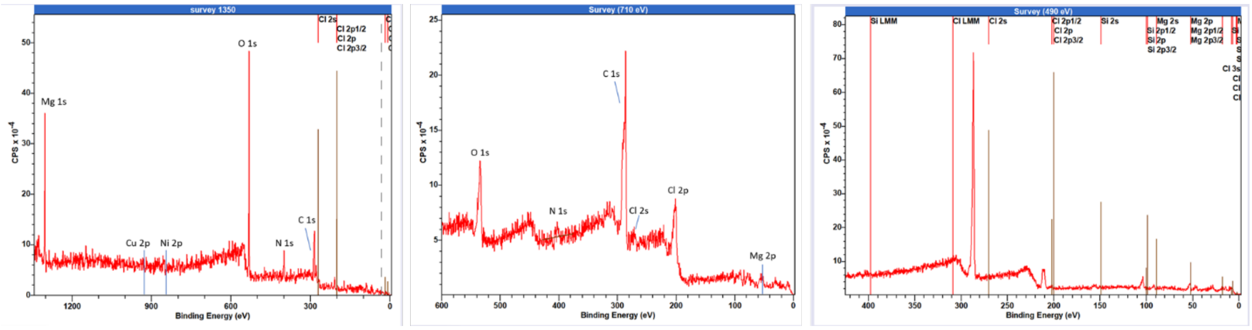

Figure S13: Depth Profiling XPS Survey Spectra- A) 9.3.1 tender x-ray survey (4000 eV photon energy) at 10-12 nm. B) 9.3.2 soft x-ray survey at 2-4 nm depth. C) 9.3.2 soft x-ray survey (490 eV photon energy) at 1-2 nm.

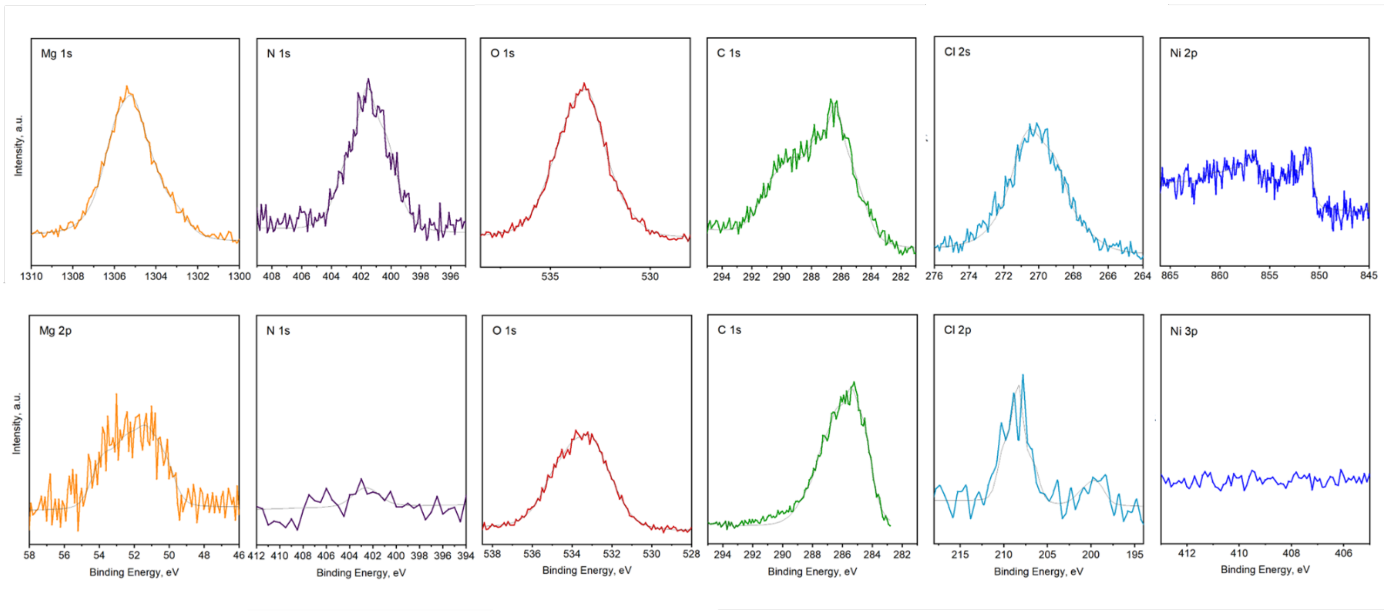

Figure S14: Depth Profiling XPS- Depth-profiling XPS spectra for Mg 1s, Mg 2p, N 1s, O 1s, C 1s, Cl 2s, Cl 2p, Ni 2p, and Ni 3p of the post-electrolysis catalyst. The top set was probed using tender X-rays at 4000 eV (10–12 nm), while the bottom set of spectra was recorded using soft X-rays at 900 eV (2–4 nm).

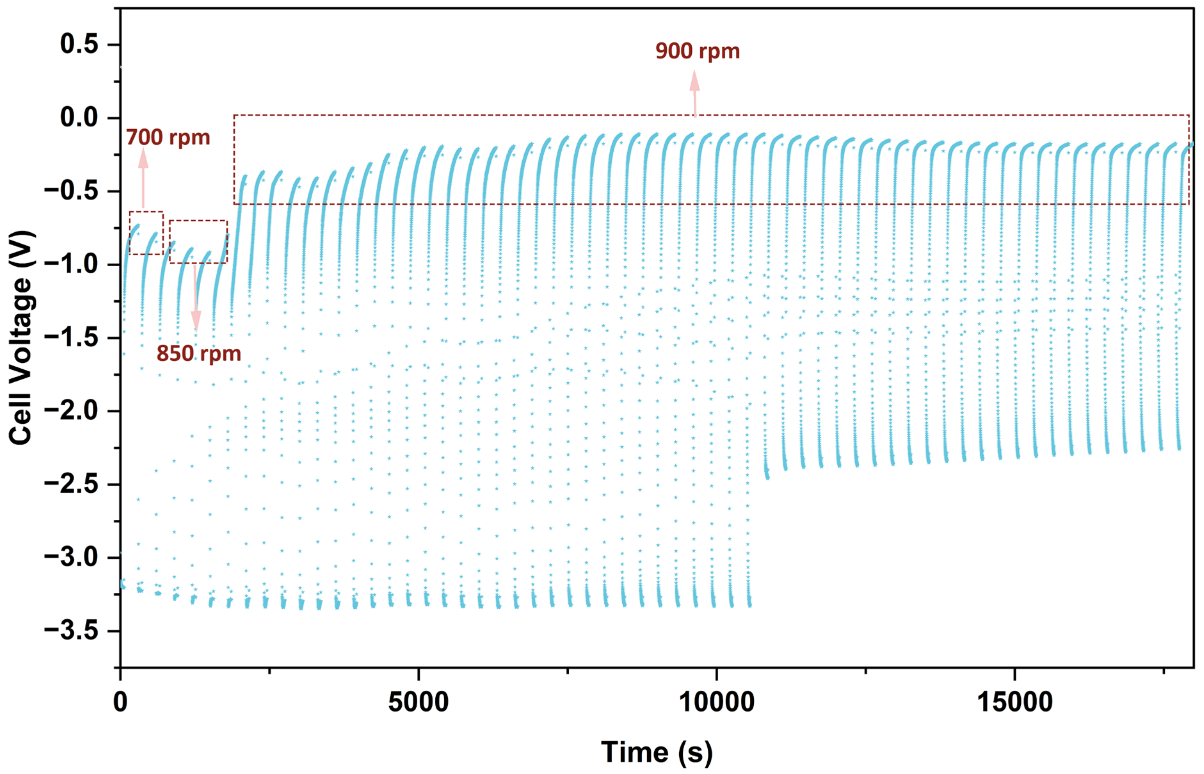

Figure S15: Effect of stirring rate on open circuit potential (OCP). The stirring rate was initially set at 700 rpm. As the OCP increased and approached -1.5 V, the stirring speed was increased to 850 rpm, which partially stabilized the potential but did not reach within the desired range of -0.1 V to -0.5 V. Further increasing the stirring rate to 900 rpm successfully restored the OCP to the target range, after which the stirring rate was maintained.

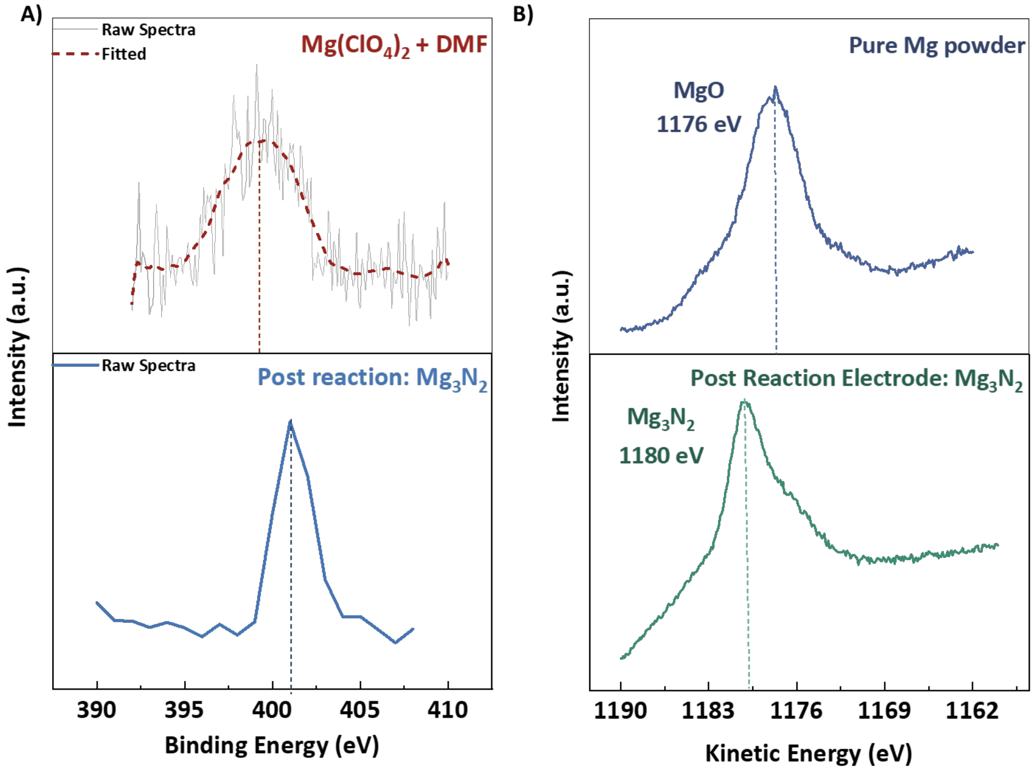

Figure S16: A) N 1s spectra showing (top) control Ni foam immersed in $Mg(ClO_4)_2$/DMF solution (2 h) and vacuum-dried (96 h), and (bottom) post-reaction electrode (96 h drying). The 2 eV shift to lower binding energy (399 eV vs. 401 eV) confirms Mg_3N_2 formation. B) Mg Auger spectra comparing (top) pristine Mg powder (showing only MgO) and (bottom) post-reaction electrode, with the characteristic Mg_3N_2 peak at 1180 eV (vs. MgO at 1176 eV).
